# On the *V*_OC_ loss in NiO-based inverted metal halide perovskite solar cells†

**DOI:** 10.1039/d4ma00873a

**Published:** 2024-10-14

**Authors:** Kousumi Mukherjee, Denise Kreugel, Nga Phung, Cristian van Helvoirt, Valerio Zardetto, Mariadriana Creatore

**Affiliations:** a Department of Applied Physics and Science Education, Eindhoven University of Technology P.O. Box 513 5600 MB Eindhoven The Netherlands k.mukherjee@tue.nl; b TNO Partner in Solliance, High Tech Campus 21 Eindhoven 5656 AE The Netherlands; c Eindhoven Institute of Renewable Energy Systems (EIRES) PO Box 513 5600 MB Eindhoven The Netherlands

## Abstract

Recent reports have shown that nickel oxide (NiO) when adopted as a hole transport layer (HTL) in combination with organic layers, such as PTAA or self-assembled monolayers (SAMs), leads to a higher device yield for both single junction as well as tandem devices. Nevertheless, implementing NiO in devices without PTAA or SAM is seldom reported to lead to high-performance devices. In this work, we assess the effect of key NiO properties deemed relevant in literature, namely- resistivity and surface energy, on the device performance and systematically compare the NiO-based devices with those based on PTAA. To this purpose, (thermal) atomic layer deposited (ALD) NiO (NiO_Bu-MeAMD_), Al-doped NiO (Al:NiO_Bu-MeAMD_), and plasma-assisted ALD NiO (NiO_MeCp_) films, characterized by a wide range of resistivity, are investigated. Although Al:NiO_Bu-MeAMD_ (∼400 Ω cm) and NiO_MeCp_(∼80 Ωcm) films have a lower resistivity than NiO_Bu-MeAMD_ (∼10 kΩ cm), the Al:NiO_Bu-MeAMD_ and NiO_MeCp_-based devices are found to have a modest open circuit voltage (*V*_OC_) gain of ∼30 mV compared to NiO_Bu-MeAMD_-based devices. Overall, the best-performing NiO-based devices (∼14.8% power conversion efficiency (PCE)) still lag behind the PTAA-based devices (∼17.5%), primarily due to a *V*_OC_ loss of ∼100 mV. Further investigation based on light intensity analysis of the *V*_OC_ and FF and the decrease in *V*_OC_ compared to the quasi-Fermi level splitting (QFLS) indicates that the *V*_OC_ is limited by trap-assisted recombination at the NiO/perovskite interface. Additionally, SCAPS simulations show that the presence of a high interfacial trap density leads to a *V*_OC_ loss in NiO-based devices. Upon passivation of the NiO/perovskite interface with Me-4PACz, the *V*_OC_ increases by 170–200 mV and is similar for NiO_Bu-MeAMD_ and Al:NiO_Bu-MeAMD_, leading to the conclusion that there is no influence of the NiO resistivity on the *V*_OC_ once interface passivation is realized. Finally, our work highlights the necessity of comparing NiO-based devices with state-of-the-art HTL-based devices to draw conclusion about the influence of specific material properties on device performance.

## Abstract 2

This study assesses the effect of key NiO properties, especially resistivity and surface energy, on the device's performance.

## Graphical abstract

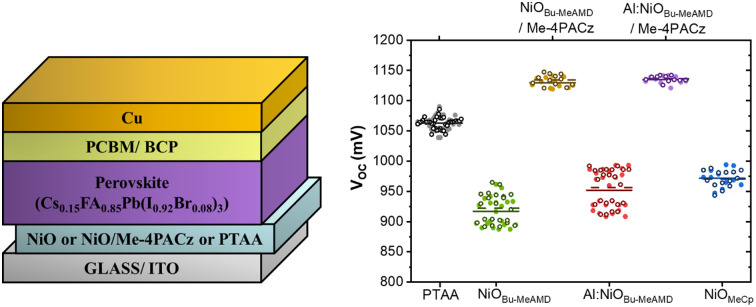

## 1. Introduction

The transition toward the production of electricity from renewable sources such as photovoltaics (PV) and wind energy is essential to solve challenges related to climate change, decrease the dependence on non-renewable sources, and strive for a sustainable future. The progress in the field of perovskite solar cells (PSCs) has been enormous with the power conversion efficiency (PCE) rising from 3.8% to 26.7% in the last 15 years.^1^ This improvement can be attributed to the engineering of the optoelectronic properties and stability of the perovskite absorber, as well as the whole device architecture, including the development of efficient and selective charge transport layers.

The inverted planar (p–i–n) architecture is particularly interesting due to lower temperature processing, enabling the application of flexible substrates in combination with a scalable fabrication process and better compatibility in monolithic tandem architecture.^2^ Various hole transport layers (HTLs) have been employed in inverted planar PSCs, ranging from organic polymers such as poly[bis(4-phenyl)(2,4,6-trimethylphenyl)amine] (PTAA),^3^ poly(3,4-ethylenedioxythiophene)–poly(styrene sulfonate) (PEDOT: PSS)^4^ and in recent years, carbazole-based self-assembling monolayers (SAMs),^5,6^ to metal oxides such as NiO^7,8^ and Cu_2_O.^9^ Devices with organic HTLs often have high PCEs but they suffer from poor environmental stability which affects the long-term stability and performance of the PSCs. Another downside is the inhomogeneous surface coverage *via* wet chemistry processing when the HTLs are processed on textured surfaces.^10^ NiO has been widely investigated in recent years. Devices with NiO have been shown to exhibit higher operational stability as compared to organic HTLs like MeO-2PACz SAM,^10^ PTAA,^11^ PEDOT:PSS,^4,12^ poly-TPD,^13^*etc.*

NiO has a wide band gap (3.4–4 eV)^8,14^ resulting in good transmittance (>80% in 400–800 nm wavelength range)^15^ and a deep valence band (5.2–5.4 eV)^14^ which aligns well with most of the metal halide perovskite absorbers. It can be deposited by solution processing,^16,17^ as well as scalable vapor deposition techniques such as sputtering^18^ and atomic layer deposition (ALD).^8,10,19^ Notably, ALD distinguishes itself from sputtering and solution processing due to its ability to grow conformal and uniform pinhole-free thin films (as low as 6 nm) even on rough, textured surfaces.^20^ It has often been adopted along with other organic HTLs in p–i–n single junction PSCs^10^ as well as in the tunnel recombination junction (TRJ) of perovskite-silicon^21^ and perovskite-CIGS tandem devices,^22^ to prevent the above-mentioned inhomogeneous surface coverage of solution-processed organic HTLs on the substrate underneath. It has also been shown to enable the homogeneous formation of SAM due to the presence of a higher concentration of hydroxyl groups than ITO, which is essential for the chemisorption of SAM.^10^ Additionally, it reduces differences in surface potential arising due to distinct microstructures of the underneath ITO film.^20,23^

Despite these advantages, champion devices using only NiO as HTL are rarely reported, mostly due to a loss in the open circuit voltage (*V*_OC_) and fill factor (FF) affecting the PCE.^8,10,22,24^ This has often been attributed to the high electrical resistivity of pristine NiO (10^4^–10^7^ Ω cm)^17,25,26^ which can affect hole transport. Decreasing the resistivity has been shown to result in efficient charge extraction and lower recombination due to reduced hole accumulation at the NiO/perovskite interface, thereby increasing the *V*_OC_.^7,27–29^ Moreover, the series resistance reduces, thereby improving the FF. For instance, Koushik *et al.* achieved a ∼40 mV *V*_OC_ and ∼9% FF gain by lowering the lateral resistivity of NiO from 870 Ω cm to 170 Ω cm by post-annealing.^8^ Doping NiO with cations such as Li,^30^ Cu,^31^ Al,^17,32,33^ Co,^34^ Ag,^35^ Zn,^36^*etc.*, also lowers NiO resistivity, improves the *V*_OC_ and modifies the band alignment of NiO HTL with respect to perovskite.

Other approaches involving physical and chemical post-treatments have also been shown to improve the bulk properties of NiO or the NiO/perovskite interface helping in improving the device performance.^8,27,37^ Modifying the surface energy of NiO has been shown to influence the growth of perovskite which can affect the perovskite morphology and the charge transport properties.^38,39^ Finally, modifying the NiO/perovskite interface by introducing an organic interlayer such as PTAA,^22^ or SAM such as [4-(3,6-dimethyl-9*H*-carbazol-9-yl)butyl]phosphonic acid (Me-4PACz),^18,40^*etc.* has also been reported to lead to a gain in *V*_OC_ and FF as compared to the NiO-only based devices.^8,10,18,22,24,41^

Very often, the above-described experimental efforts do not include a comparison between NiO-only-based devices and state-of-the-art organic HTL-based devices. The lack of comparison makes it challenging to draw conclusions about the efficacy of the chosen approach to improve the bulk and/or surface properties of NiO towards high-efficiency devices. Therefore, in this study, we have investigated three different ALD NiO HTLs with variable lateral resistivity and surface energy and have compared the NiO-based device's performance to PTAA-based devices. For this purpose, a novel ALD process to dope NiO with aluminium using the sequential precursor approach is reported in this work. Al is chosen to dope the most resistive NiO as it leads to a decrease in NiO resistivity without affecting its transmittance, unlike other dopants such as Co,^42^ Li,^43,44^*etc.* We observe that lowering the resistivity of NiO results in a slight *V*_OC_ gain of ∼30 mV, with respect to the most resistive NiO-based device. However, all NiO-based devices have an average PCE of ∼14.8% and they lag behind the PTAA-based devices due to a major *V*_OC_ loss of ∼100 mV. This *V*_OC_ loss is recovered by surface modification of NiO with Me-4PACz, thereby attributing the lower performance to the quality of the NiO/perovskite interface. Light intensity dependence of photovoltaic parameters, time-resolved photoluminescence (TR-PL) and absolute photoluminescence point out that interface recombination phenomena limit hole extraction from the perovskite to NiO, thereby leading to the *V*_OC_ loss. Furthermore, SCAPS (solar cell capacitance simulator) simulation is carried out to validate our experimental results and show the effect of interfacial defect states on the device and the *V*_OC_. We also argue that the conclusions drawn so far in literature on the beneficial role of the NiO conductivity on the device performance^17,33,45–47^ can be shadowed by the interface loss phenomena between NiO and perovskite.

## 2. Methods

### 2.1. Atomic layer deposition of NiO

The undoped and aluminium-doped thermal ALD NiO processes (hereafter, referred to as NiO_Bu-MeAMD_ and Al:NiO_Bu-MeAMD_ respectively) are carried out in the commercial FlexAL™ MK1 (Oxford Instruments) ALD reactor and the plasma-assisted ALD NiO process (hereafter, referred to as NiO_MeCp_) is developed in the home-built ALD reactor previously introduced.^8,20^ Both ALD reactors are high vacuum systems equipped with a rotary and turbomolecular pumping unit that can reach a base pressure of 10^−6^ Torr. All the processes are carried out at a deposition temperature of 150 °C. The detailed process description of the NiO_Bu-MeAMD_ and NiO_MeCp_ and the ALD process development of the Al:NiO_Bu-MeAMD_ are mentioned in Section S1 (ESI†) and summarised in Table 1.

**Table 1:** Overview of the different ALD processes used in this work. GPC refers to the growth per cycle of the ALD process

ALD process	ALD precursor and co-reactant	Deposition temperature (°C)	GPC (Å/cycle)	Ref.
NiO_Bu-MeAMD_	(*N*,*N*′-di-*tert*-butylacetamidinato)Nickel(ii) + water	150	0.43 ± 0.01	20
Al:NiO_Bu-MeAMD_	NiO: (*N*, *N*′-di-*tert*-butylacetamidinato)Nickel(ii) + water	150	0.44 ± 0.02	Developed in this work
	Al-dopant: dimethyl aluminium isopropoxide + water			
NiO_MeCp_	bis-methylcyclopentadienyl nickel + oxygen plasma	150	0.29 ± 0.01	8 and 48

### 2.2. NiO film characterization

The thickness of the NiO films is determined by spectroscopic ellipsometry (SE) (NIR Ellipsometer M2000, J.A. Woollam Co.). For this purpose, NiO film is deposited on c-Si (100) substrates, and the growth is monitored by *in situ* SE (VIS Ellipsometer M200, J.A. Woollam Co.). The ellipsometry spectra of the NiO are modelled by a Cauchy dispersion model in the wavelength range of 1.25–3.5 eV. The SE data of the c-Si and the native SiO_2_ are modelled according to the model reported by Herzinger *et al.*^49^ The elemental composition of the NiO films is analyzed by Rutherford backscattering spectroscopy (RBS) and elastic recoil detection (ERD) using a 3.5 MV Singletron with a 1000 keV He+ beam at an angle almost perpendicular to the sample (170°) and also a glancing angle. For this purpose, the Al:NiO_Bu-MeAMD_ film is deposited on glassy carbon substrates with 50 nm Ti oxide on top instead of c-Si substrates to prevent overlapping of the RBS spectrum of Al with Si substrate. Furthermore, X-ray photoelectron spectroscopy (XPS) is carried out in the Thermo Scientific KA1066 spectrometer using monochromatic Al Kα X-rays with an energy of 1486.6 eV and without any pre-sputtering. The data is analyzed by the Avantage software with a Smart background (Shirley background with offset correction) subtraction. The results are corrected for sample charging using the C1s orbital as a reference with a binding energy of 284.8 eV. The transmittance of the NiO films is characterized using ultraviolet-visible (UV-Vis) spectrophotometry using a PerkinElmer LAMBDA 1050 spectrophotometer. The measurement is carried out inside an integrating sphere by exciting it from the glass/ITO side. The crystal structure of the NiO films on c-Si substrates is analyzed by X-ray diffraction (XRD) in a Bruker D8 discover using a Cu kα (*λ* = 1.54 Å) radiation in the range of 30°–80° with a step size of 0.04° and a time per step of 10 s in the grazing incidence (GI) mode with an incidence of 0.4°. The contact angle of different liquids on the NiO surface is measured using a contact angle goniometer (Ramé-Hart Incorporation). The surface energy of the NiO is calculated by the Owens–Wendt–Rabel and Kaelble Model using 3 liquids with different ratios of the polar and dispersive components- ethanol, water, and ethylene glycol. The lateral resistivity of the NiO films is measured at room temperature using a Signatone four-point probe in combination with a Keithley 2400 source meter, right after deposition. For this purpose, the films are deposited on c-Si/450 nm SiO_2_ substrates.

### 2.3. Solar cell fabrication

Patterned soda-lime glass/indium tin oxide (ITO) (BBBB configuration, 3 × 3 cm^2^) from Naranjo is used for the single junction PSCs. These substrates are first cleaned in soap water, de-ionized water, and isopropanol (IPA) in subsequential steps for 15 minutes in an ultrasonic bath at room temperature. They are left in IPA overnight followed by an additional cleaning of 15 minutes in an ultrasonic bath. Then, they are dried with an N_2_ gun. Next, the hole transport layer (HTL) – PTAA or NiO, is deposited. For the PSCs with poly(triarylamine)(PTAA) as the HTL, a 2 mg mL^−1^ solution of PTAA (Solaris Chem) in toluene (Sigma- Aldrich) is spin-coated on the ITO at 5000 rpm for 35 s followed by annealing at 100 °C for 10 minutes. The ALD processes for NiO_MeCp_, Al:NiO_Bu-MeAMD_ and NiO_Bu-MeAMD_ are described above. The resultant film thickness for the Al:NiO_Bu-MeAMD_ with a cycle ratio ‘2*m*:1’ =135 is ∼11 nm and the film thicknesses of the undoped NiO_Bu-MeAMD_ and NiO_MeCp_ are also kept at ∼11 nm for a fair comparison. For the NiO-based PSCs with the [4-(3,6-dimethyl-9*H*-carbazol-9-yl)butyl]phosphonic acid (Me-4PACz) SAM, a 1 mM Me-4PACz (Tokyo Chemical Industry, >99.0%) solution is prepared by dissolving it in anhydrous ethanol (Sigma-Aldrich), followed by sonication in an ultrasonic bath for 30 minutes for full dispersion. The Me-4PACz SAM is deposited on NiO_Bu-MeAMD_ and Al:NiO_Bu-MeAMD_*via* static spin-coating by dropping 120 μL of the solution and waiting for 90 s for the solution to spread, followed by 30 s of spin-coating at 3000 rpm. Then, the layers are annealed at 100 °C for 10 minutes.

In this study, a dual-cation perovskite Cs_0.15_FA_0.85_Pb(I_0.92_Br_0.08_)_3_ solution is used. The procedure followed is reported by Bracesco *et al.*^50^ In short, it is prepared by mixing a stoichiometric amount of the following precursors: PbI_2_ (Alfa Aesar, 99.999%) and PbBr_2_ (Tokyo Chemical Industry, 99.9%), FAI (Greatcell (Dyesol), 99.9%), and CsI (Alfa Aesar, 99.9%), in anhydrous DMF: DMSO (Sigma-Aldrich, >99.9%) (9 : 1 volume ratio), to achieve a concentration of 1.33 M. Then, the perovskite solution is stirred overnight at room temperature. It is spin-coated with a two-step procedure: at 2000 rpm for 10 s and 5000 rpm for 30 s. During the first step, 110 μL precursor solution is dynamically spin-coated on the substrate 5 s after the start of the program. Next, 250 μL chlorobenzene (Sigma-Aldrich) anti-solvent is dropped on the spinning substrate 21 s after the start of the program to quench the film and form a smooth and compact layer. The film is subsequently annealed on a hotplate at 100 °C for 10 min.

Phenylethylammonium iodide (PEAI, Sigma Aldrich, 98%) is used for passivating the ETL- perovskite interface, in some devices (mentioned in the text), by spincoating 100 μL of 1.5 mg mL^−1^ of PEAI solution in isopropanol, at 3000 rpm for 45 s followed by annealing at 100 °C for 10 min. For the electron transport layer (ETL), a 20 mg mL^−1^ PCBM (Solenne B.V., 99%) solution in chlorobenzene is spin-coated onto the perovskite layer at 1500 rpm for 50 s. This is followed by the spin-coating of 1 mg mL^−1^ bathocuprine (Sigma-Aldrich, 96%), in short BCP, solution in ethanol (SigmaAldrich) at 5000 rpm for 30 s. Finally, the 100 nm Cu electrode is deposited by thermal evaporation of Cu (Kurt J. Lesker Co.) at a pressure of 10^−6^ mbar using a shadow mask, defining an active device pixel area of 0.09 cm^2^.

### 2.4. Device characterization

The current density–voltage (*J*–*V*) measurements are carried out in a WACOM solar simulator which is calibrated with a Si reference cell from Fraunhofer ISE to simulate the AM1.5 spectrum. The cell area of 0.09 cm^2^ is defined by a stainless-steel shadow mask. The scan speed is 200 mV s^−1^ with a step size of 20 mV controlled by a Keithley 2400 source-measure unit. The maximum power point tracking (MPPT) is performed for 3 minutes. The light intensity-dependent *J*–*V* measurements are carried out in a nitrogen-filled glovebox under a white light halogen lamp. The light intensity is calibrated to 100 mW cm^−2^ using a silicon reference cell to simulate the AM 1.5 spectrum. A set of neutral filters are used to obtain different intensities of 1, 0.83, 0.53, 0.33, 0.1, 0.01, and 0.001 suns. A Keithley 2400 is used to measure the *J*–*V* curves with a scanning rate of 200 mV s^−1^ with a 20 mV step. The EQE measurements are performed using a home-built modulated monochromatic probe light to illuminate the solar cell. Absolute photoluminescence (PL) is measured using a 455 nm fibre-coupled LED (Thorlabs) source with an intensity of 1 sun to excite the perovskite film through an optical fibre placed in an integrating sphere (Avantes, AvaSphere30-REFL) fitted with a 550 nm short-pass filter (Edmund Optics). The spectrum is measured through an optical fibre connected to a calibrated spectrometer (Avantes, AvaSpec-HERO) using a 550 nm long-pass filter.

The time-integrated photoluminescence (PL) and time-resolved photoluminescence (TRPL) measurements of the perovskite deposited on the ITO/NiO (or PTAA) are carried out in a home-built setup. The samples are excited using a laser with an excitation wavelength of 420 nm, a repetition frequency of 5 MHz, and an excitation power of around 35 μW. The spot size is around 83 ± 8 μm and the fluence is around 128 ± 13 nJ cm^−2^. The structural properties of the perovskite film are analyzed by XRD in the Bragg-Brentano mode in the full range, *i.e.* 11.4° to 55° with a step size of 0.01° and a time per step of 0.2 s. GI-XRD measurements are carried out in the range of 11.4° to 16° with a step size of 0.05° and a time per step of 10 s with an incidence angle of 1° to check the presence of PbI_2_. The morphology is checked by scanning electron microscopy (SEM) imaging carried out using a JEOL JSM-7500FA. The images are made using an accelerating voltage of 1.5–2 kV and a probe current of 7 μA.

## 3. Results and discussions

All three NiO films – NiO_Bu-MeAMD_, Al:NiO_Bu-MeAMD_ and NiO_MeCp_, are slightly oxygen-rich with a Ni/O ratio in the range of 0.88–0.96 ± 0.03. This is consistent with what has been reported in the literature so far.^13,51^ The concentration of Al, as quantified from RBS (Table S1, ESI†), in the Al:NiO_Bu-MeAMD_ film is 0.9 at%. A detailed characterization of these NiO films can be found in Section S2 (ESI†). The lateral resistivity of the NiO films along with other key material properties discussed later in the study, are reported in Table 2. The resistivity of the NiO_Bu-MeAMD_ is nearly a factor of 200 higher than NiO_MeCp_. It decreases to ∼400 Ω cm upon doping with Al, as it creates shallow acceptor states at low Al at% (<2.5%).^52,53^ The work function, reported in Table 2, is however not affected by Al-doping because the Al-rich region in Al:NiO_Bu-MeAMD_, is buried under a NiO layer according to the supercycle approach and the UPS, with a penetration depth of ∼2 nm, is sensitive to the NiO top layer. The energy difference between the VBM and W.F. decreases from 0.87 ± 0.07 eV for the NiO_Bu-MeAMD_ to 0.75 ± 0.01 eV upon doping it with Al.

**Table 2:** Bulk and surface material properties of NiO_Bu-MeAMD_, Al:NiO_Bu-MeAMD_ and NiO_MeCp_. The resistivity data was determined from 5 samples each of NiO_Bu-MeAMD_ and Al:NiO_Bu-MeAMD_ and 15 samples of NiO_MeCp_. The mass density of the NiO is calculated from RBS measurements and the thickness derived from spectroscopic ellipsometry analysis

	NiO_Bu-MeAMD_	Al:NiO_Bu-MeAMD_	NiO_MeCp_
Resistivity (Ω cm)	(1.7 ± 0.3) × 10^4^	430 ± 97	70 ± 20
Preferred crystal orientation	(111)	(111)	(200)
Surface energy (mN m^−1^)	91 ± 1	85 ± 1	50 ± 1
Transmittance (%) (400–800 nm)	84	85	82
Density (g cm^−3^)	5.1 ± 0.3	4.5 ± 0.3	7.0 ± 0.7
Work function (eV)	4.47 ± 0.05	4.47 ± 0.05	4.70 ± 0.05
Valence band maximum (eV)	5.34 ± 0.05	5.21 ± 0.05	5.46 ± 0.05

**Fig. 1:**
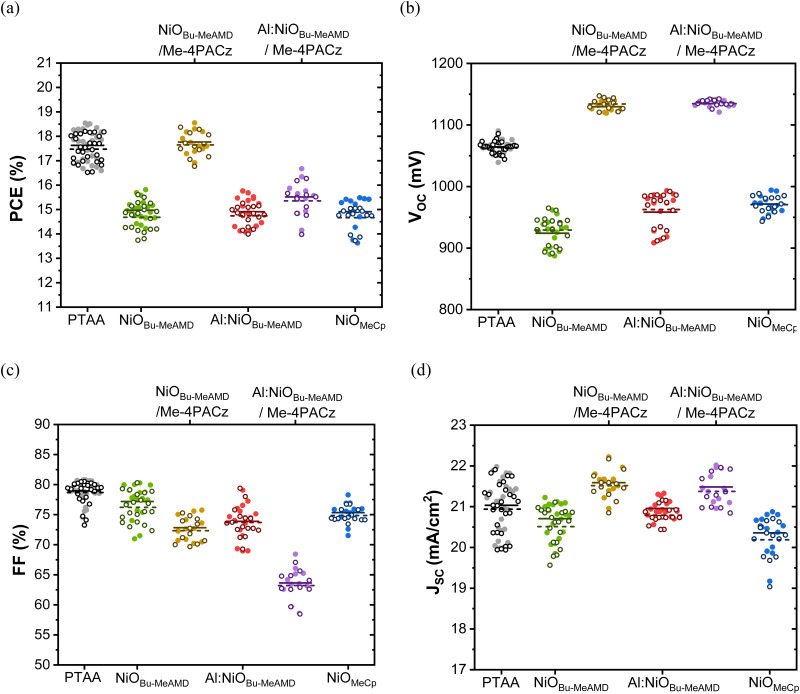
PV parameters of the different HTL-based devices (a) PCE, (b) *V*_OC_, (c) FF, and (d) *J*_SC_. The filled circles and the solid line represent the reverse *J*–*V* scan whereas the empty circles and the dashed line represent the forward *J*–*V* scan. The solid and dashed lines represent the mean value of the PV parameter during the reverse and forward scans respectively. These data are collected from several batches on 30 devices with PTAA, 20 devices with NiO and 15 devices with NiO/SAM.

In this study, we have adopted a p–i–n architecture consisting of glass/ITO/NiO/Cs_0.15_FA_0.85_Pb(I_0.92_Br_0.08_)_3_/PCBM/BCP/Cu and compared it to a reference device with the conventional PTAA as HTL. Fig. 1 and Table 3 report the *J*–*V* parameters of the devices. The devices with undoped NiO_Bu-MeAMD_ yield a PCE of ∼14.8%, with a *J*_SC_ of ∼20.6 mA cm^−2^, an FF of ∼77%, and a *V*_OC_ of ∼940 mV. There is a gain of ∼30 mV in *V*_OC_ for Al:NiO_Bu-MeAMD_ and NiO_MeCp_-based devices, likely due to the decrease in resistivity when compared to the NiO_Bu-MeAMD_. The improvement in *V*_OC_ is in line with what has been observed in literature.^8,17,27,33^ There is also a slight gain in *J*_SC_ for Al:NiO_Bu-MeAMD_ whereas the *J*_SC_ decreases to ∼20.3 mA cm^−2^ for NiO_MeCp_-based devices. The difference in *J*_SC_ is corroborated by the integrated current (*J*_SC,EQE_) calculated from the EQE spectra obtained for the different HTL-based devices, as shown in Fig. S7 (ESI†) and reported in Table S2 (ESI†). The slight loss in the *J*_SC_ observed for the NiO_MeCp_-based devices, as compared to those based on NiO_Bu-MeAMD_ and Al:NiO_Bu-MeAMD_, is confirmed by the lower *J*_SC,EQE_. This also corresponds to the slightly lower transmittance of NiO_MeCp_, as shown in Fig. S4 (ESI†).

**Table 3:** Average values of PV parameters, shunt (*R*_shunt_) and series resistance (*R*_series_) along with their standard deviation of the different HTL-based PSCs. The *R*_shunt_ and *R*_series_ are calculated from the slope of the *J*–*V* curve at the *J*_SC_ and *V*_OC_ respectively. This data is collected from several batches on 30 devices with PTAA and 20 devices with NiO

	V_OC_ (mV)	J_SC_ (mA cm^−2^)	FF (%)	PCE (%)	R_series_ (Ω cm^2^)	R_shunt_ (kΩ cm^2^)
NiO_Bu-MeAMD_	937 ± 33	20.6 ± 0.4	77 ± 3	14.8 ± 0.6	4.0 ± 0.5	2.7 ± 1.2
Al:NiO_Bu-MeAMD_	961 ± 28	20.9 ± 0.2	74 ± 3	14.8 ± 0.5	4.7 ± 0.7	2.9 ± 0.7
NiO_MeCp_	971 ± 14	20.3 ± 0.5	75 ± 1	14.8 ± 0.5	3.9 ± 0.4	2.4 ± 0.4
PTAA	1064 ± 10	20.9 ± 0.6	79 ± 2	17.5 ± 0.7	3.7 ± 0.3	3.8 ± 1.2

Despite the lower resistivity of the Al:NiO_Bu-MeAMD_ and NiO_MeCp_, their corresponding devices suffer from a FF loss, specifically up to 3% absolute value with respect to the NiO_Bu-MeAMD_-based devices. Thus, in the case of Al:NiO_Bu-MeAMD_, the overall gain in *J*_SC_ and *V*_OC_ balances the loss in FF, resulting in a PCE of ∼14.8%. The NiO_MeCp_-based devices also exhibit PCE similar to the Al:NiO_Bu-MeAMD_ and NiO_Bu-MeAMD_. The FF loss observed in the Al:NiO_Bu-MeAMD_ and NiO_MeCp_-based devices can be due to the higher *R*_series_ and lower *R*_shunt_ (see. Table 3), respectively when compared to the NiO_Bu-MeAMD_-based devices. Studies have shown that especially at a low *R*_shunt_ (<10^5^ kΩ cm^2^), a small increase in *R*_series_ or a decrease in *R*_shunt_ can lead to a loss of 1–3% in FF.^54,55^ Here, it should be noted that *R*_series_ depends on the resistance of the individual layers as well as the interfacial contact resistance between the layers. The presence of hydroxyl groups has been shown to increase the interface contact resistance between NiO and perovskite.^56^ We have detected hydroxyl groups on NiO surface based on ERD measurements (see, Table S1, ESI†) and the O1s spectra (Fig. S3(b), ESI†). The H present in metal oxides is in the form of hydroxyl groups and thus, the H content in the films provides information on the hydroxyl content.^57,58^ The Al:NiO_Bu-MeAMD_ layer has a higher concentration of hydroxyl groups (∼13 at. nm^−3^ as compared to ∼10 at. nm^−3^ for NiO_Bu-MeAMD_) and in parallel, the OH feature contribution at 531 eV in the O1s spectra confirms such a difference between the 2 layers. Since the Al:NiO_Bu-MeAMD_ layer has a lower resistivity than NiO_Bu-MeAMD_ and we observe no influence of the NiO properties on the perovskite bulk properties, specifically crystallinity and morphology (discussed later), we argue that the larger (relative) concentration of hydroxyl groups on the Al:NiO_Bu-MeAMD_ surface leads to an increase in interface contact resistance with the perovskite.

When compared with NiO-based PSCs, PTAA-based PSCs exhibit a higher PCE of ∼17.5%. The *J*_SC_ of the PTAA-based PSCs is similar to the NiO-based PSCs but the FF is slightly higher by 2–5% in absolute value. The *R*_shunt_ of the PTAA-based devices is slightly higher and its *R*_series_ is slightly lower than the NiO-based devices, resulting in the increase in FF. From this, we conclude that the performance of the NiO-based devices lags behind those based on PTAA, despite decreasing NiO film resistivity, due to a significant *V*_OC_ loss in the range of 90–130 mV.

**Fig. 2:**
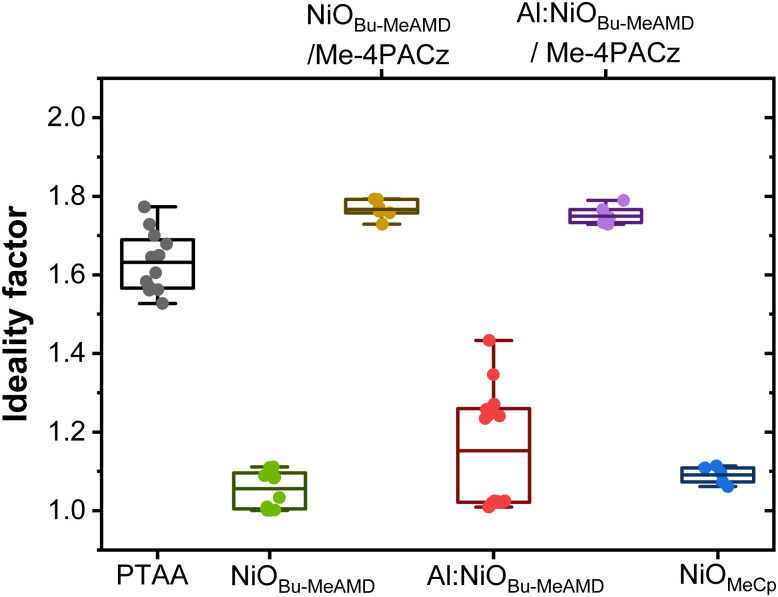
Ideality factor of different HTL-based devices (without PEAI passivating the ETL/perovskite interface), inferred from the slope of the *V*_OC_*vs.* light intensity graph. The box represents 25–75% percentile of the distribution with the mean as the middle line. The whiskers are 5 and 95% percentile of the distribution and the rest are outliers.

The ideality factor is calculated from the light intensity dependence of the *V*_OC_^59^ to gain insight into the dominant recombination mechanism affecting the *V*_OC_. The PTAA-based devices have an average ideality factor of ∼1.6 (Fig. 2) indicating that Shockley–Read–Hall (SRH) recombination within the perovskite bulk is the dominant recombination mechanism. For all the NiO-only-based devices, the ideality factor is in the range of 1.1–1.3. It has been previously noted that the ideality factor is not always a superposition of the SRH and bimolecular recombination, but it is also affected by interface recombination, especially in devices with low PCE and *V*_OC_.^60,61^ Moreover, when we take care of passivating the other interface *i.e.* the ETL (PCBM)/perovskite interface by PEAI, the ideality factor still remains around 1.1 (Fig. S8, ESI†). This suggests that the NiO- perovskite interface is non-ideal and limits the device performance.

At the same time, we observe that the *V*_OC_ loss in the NiO-based devices is recovered after modifying the NiO/perovskite interface by the Me-4PACz SAM interlayer (see, Fig. 1(b)). The increase in the *V*_OC_ hints at better charge extraction induced by Me-4PACz. Similar large improvement in *V*_OC_ (100–200 mV) has also been observed in the literature when using a carbazole-based SAM interlayer between NiO and perovskite, irrespective of the deposition method of NiO.^10,62–66^ The ideality factor of these devices also increases to ∼1.8 indicating that interface recombination is mitigated and that SRH charge recombination primarily occurs in the bulk perovskite. Therefore, we confirm that the reason behind the loss in NiO-only-based device performance is interface recombination and any beneficial effect from the decrease in NiO resistivity may be shadowed by phenomena at interfaces. The role of the SAM will be discussed in detail later in the manuscript.

Next, we analyze the potential causes behind the poor quality of the NiO/perovskite interface. We first investigate the crystallographic properties of NiO and its surface chemistry and their effect on the perovskite morphology and bulk properties. The crystallographic properties of NiO films, as determined by GI-XRD, are shown in Fig. S5 (ESI†). The preferential crystal orientation of undoped and Al:NiO_Bu-MeAMD_ is (111) while it is (200) for NiO_MeCp_. The crystal orientation of the NiO has been shown to influence the surface energy of the film with the (111) orientation being polar in nature.^67^ This results in a higher surface energy (and higher hydrophilicity) of the Al:NiO_Bu-MeAMD_ and NiO_Bu-MeAMD_ than NiO_MeCp_, (see, Table 2). However, no visible difference in the wettability of the perovskite solution during spin-coating as well as of the film coverage is observed on the different NiO films. Top-view SEM images in Fig. S9 (ESI†), show that there is only a small difference in the average grain size of the perovskite grown on hydrophobic PTAA and NiO_MeCp_ (grain in the diameter range of ∼160–170 nm) and Al:NiO_Bu-MeAMD_ and NiO_Bu-MeAMD_ (both in the range of ∼190–200 nm). All perovskite films are without voids and pinholes, therefore the presence of local shunt pathways is excluded. Furthermore, no bright needle-shaped grains signifying the presence of lead iodide (PbI_2_) are observed on top of the perovskite layers which could have been from unreacted precursor material or due to storage and exposure.^68^

**Fig. 3:**
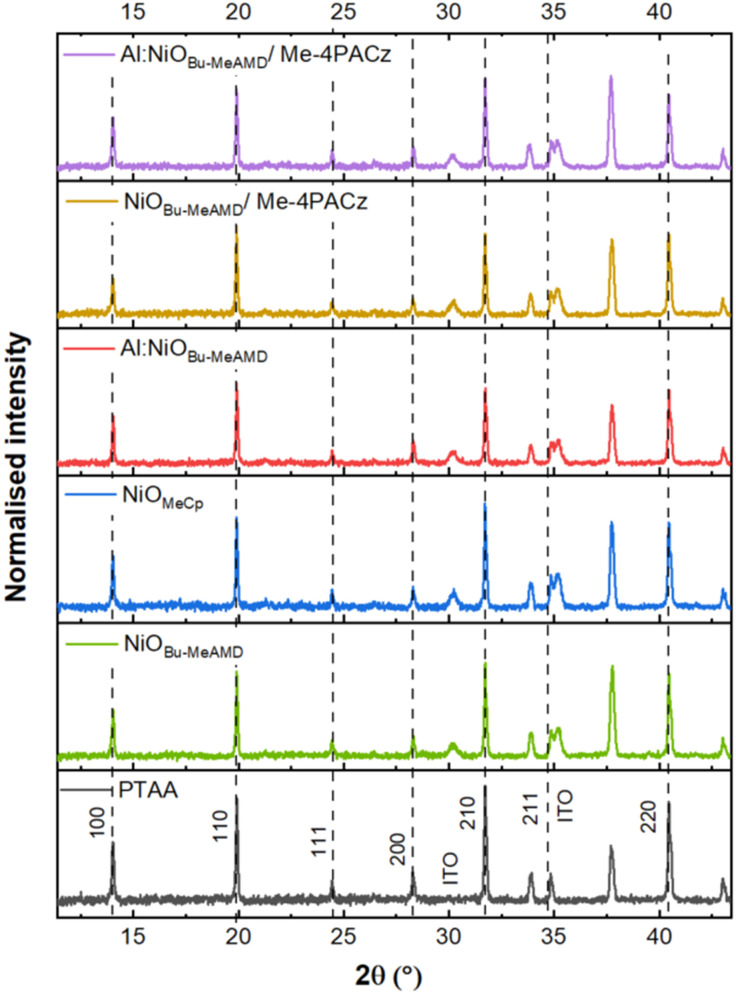
XRD pattern of perovskite layers deposited on different HTLs.

XRD measurements are carried out on the ITO/NiO (or PTAA)/perovskite/tri-octyl phosphine oxide (TOPO) stack to seek any difference in the bulk crystallographic properties of the perovskite processed on top of the different HTLs. The thin layer of TOPO is spin-coated on top of the stack to limit exposure to ambient air. As shown in Fig. 3, the main diffraction peaks of the perovskite films are at 14.0°, 19.9°, 28.3°, 31.7°, 34.8° and 40.5° corresponding to the (100), (110), (200), (210), (211) and (220) crystal planes, respectively.^69^ The peaks at 30° and 35° can be assigned to the ITO^70^ and the peak at 37.5° is a contribution of the XRD sample holder. There is a negligible difference in the XRD peak positions and intensity for the different HTL-based stacks, indicating that the perovskite crystallographic quality is not affected by the surface properties of the HTLs underneath. In conclusion, the surface energy of NiO is not expected to be the cause of the *V*_OC_ loss addressed earlier because it has no effect on the perovskite solution wettability and its bulk properties.

The presence of PbI_2_ in NiO-based devices has been reported in literature to lead to a *V*_OC_ loss in the devices. This has often been attributed to a redox reaction at the NiO/perovskite interface between the Ni^3+^ sites and the organic A-site cation of perovskite.^13,71^ Also, it has been shown that PbI_2_ is formed due to the decomposition of perovskite when it is in direct contact with the hydroxyl groups on the NiO surface.^72–74^ Boyd *et al.* suggested that the formation of the PbI_(2−*x*)_Br_*x*_ compound can impede charge extraction by NiO and result in *V*_OC_ loss.^13^ Interestingly, in our study, we do not consistently observe the PbI_2_ peak at ∼12.6° across our several batches. XRD measurement carried out after a month to evaluate whether the NiO/perovskite stack undergoes a pronounced degradation upon ageing in an inert atmosphere,^75^ shows that PbI_2_ is not consistently present in all the different NiO-based stacks, as shown in Fig. S10 (ESI†), *i.e.* it is absent in the NiO_MeCp_-based half stack, whereas it is present in the other NiO-based stacks. Moreover, we detect PbI_2_ also in the case of NiO_Bu-MeAMD_/Me-4PACz and PTAA-based stack. GIXRD measurements (Fig. S10(b), ESI†) carried out on the same stacks at an incidence angle of 1° to solely probe the perovskite bulk, indicate that this PbI_2_ is present in the bulk of the perovskite. From this, we would like to point out that Brag-Brentano XRD measurements, generally reported in literature studies,^13,71^ is not sufficient to conclude whether PbI_2_ is present in the bulk or at the interface. Instead, varying the incidence angle of X-rays in GIXRD measurements can be useful to probe different penetration depths in perovskite.

**Fig. 4:**
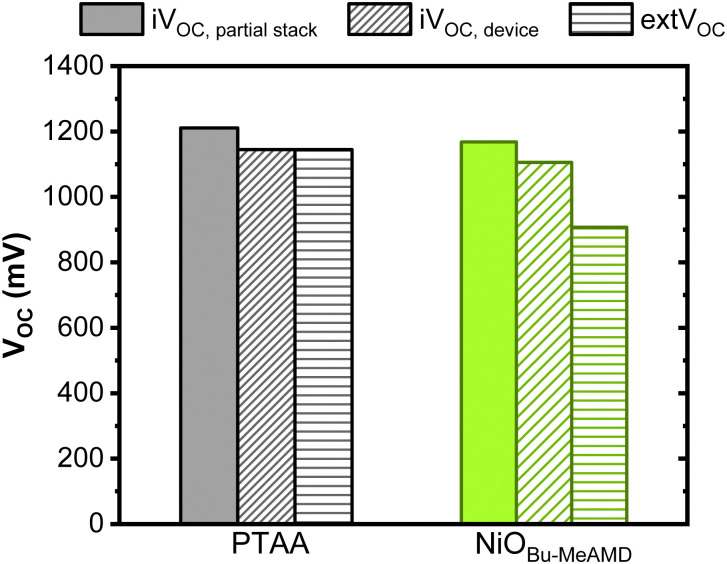
QFLS (implied *V*_OC_) of the PTAA and NiO_Bu-MeAMD_-based partial stacks and devices calculated from absolute PL and compared with the externally measured *V*_OC_.

We further look into absolute photoluminescence (PL) measurements to quantify the non-radiative recombination loss calculated from the quasi-Fermi level splitting (QFLS) of the devices and elucidate the reason behind this loss. For this purpose, only the NiO_Bu-MeAMD_ and PTAA-based devices are compared. The QFLS of the NiO-based partial stack has a 40 mV loss with respect to the PTAA-based stack indicating that the latter forms a better interface with the perovskite (Fig. 4). Addition of the ETL and the top electrode to the partial stack results in a ∼60 mV loss in the implied *V*_OC_ in both NiO and PTAA-based devices which can be due to non-radiative recombination at the perovskite/PCBM interface. The implied *V*_OC_ from the QFLS for the PTAA-based devices coincides with the externally measured *V*_OC_. This shows that the QFLS and externally measured *V*_OC_ are governed by the same recombination mechanism. On the other hand, the devices with NiO_Bu-MeAMD_ suffer from ∼200 mV loss in *V*_OC_ with respect to the implied *V*_OC_. This can be due to a band misalignment between the valence band levels and/or fast interface recombination.^60,76^ There is no indication of a band misalignment for the NiO/perovskite as the valence band maximum (VBM) of the NiO_Bu-MeAMD_ is 5.3 eV (see, Table 2 and Fig. S6, ESI†) and is similar to that of PTAA (5.20 eV^77^). On the other hand, a high interface recombination rate at the NiO/perovskite interface can deplete hole density near this interface resulting in asymmetric band bending of the hole quasi-Fermi level (‘*E*_F,hole_’) in the perovskite absorber and in turn, leading to a decrease in the *V*_OC_ of the NiO-based devices.^60,76^ The decrease in implied *V*_OC_ indicates that a high rate of interface recombination affects the NiO-based devices.

**Fig. 5:**
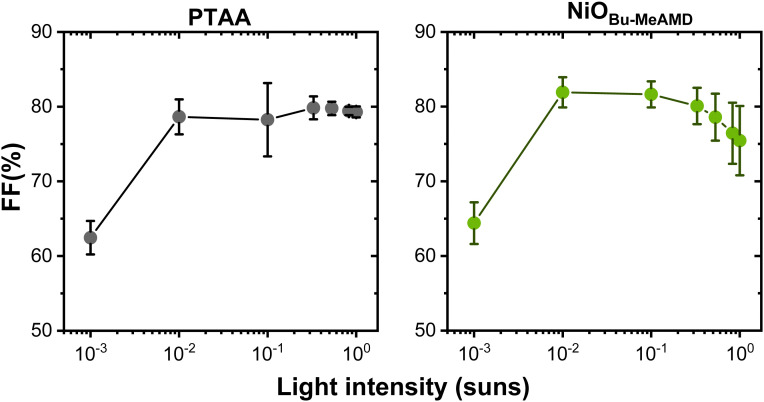
FF *vs.* light intensity of PTAA and NiO_Bu-MeAMD_-based devices. The average and the standard deviation are based on 8 devices.

The light intensity dependence of the FF is analysed to provide further insight into the charge carrier transport and recombination mechanisms. Fig. 5 shows the changes in FF at high (1 sun) and low (10^−4^ sun) intensities compared to the maximum FF obtained at 10^−2^ sun intensity. The FF decreases drastically at low illumination for all the devices indicating a case of low shunt resistance. This is also supported by the fact that both NiO and PTAA-based devices have low shunt resistance (<10^5^ Ω cm^2^).^55^ The maximum FF at 10^−2^ sun of the NiO, as well as of the PTAA-based devices, is lower than the Shockley Queisser limit (∼90%^78^) suggesting that trap-assisted recombination in the bulk perovskite absorber can affect the device performance. The FF decrease in the NiO_Bu-MeAMD_-based devices at both low and high intensities compared to the maximum value at 10^−2^ sun indicates that they suffer from a combination of ohmic and trap-assisted interfacial recombination losses.^79^ At a high light intensity, a higher concentration of charge carriers is generated and there is an asymmetry in the non-radiative recombination at the interface with higher trap sites resulting in the FF drop at high intensities.^41,79^ Previously, Glowienka *et al.* have also reported a similar trend in the FF with varying light intensities and showed *via* simulation based on the drift-diffusion model that there is an asymmetric band-bending at the NiO/perovskite interface.^41^ Hence, based on the above discussions, we conclude that NiO-based devices have a higher recombination rate at the NiO/perovskite interface due to the presence of traps as compared to the PTAA-based devices. This can result in the *V*_OC_ loss observed in the NiO-based devices. We believe that the presence of Ni^3+^ states in our NiO films, shown in the Ni2p spectra in Fig. S3 (ESI†), can act as recombination centres at the NiO-perovskite interface, as has also been reported by Wang *et al.*^80^ Ni^3+^ states are electron acceptors (see, Section S2, ESI†) and therefore, it can also prevent hole extraction when present on the NiO surface.^24^

**Fig. 6:**
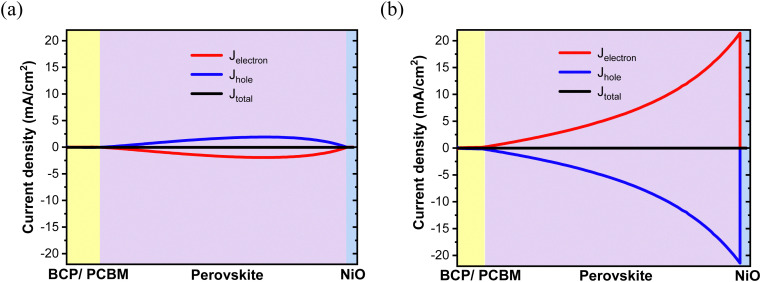
Simulated electron current density (*J*_electron_), hole current density (*J*_hole_) and total current density (*J*_total_) of (a) NiO-based devices with no interface defect states, and (b) NiO-based devices with 10^10^ cm^−2^ defect states at the NiO/perovskite interface, at open circuit condition.

Next to the experimental results, we have carried out simulations *via* the solar cell capacitance simulator software (SCAPS)^81^ to correlate the loss in *V*_OC_ with the presence of NiO/perovskite interface defect states. The parameters used for the simulation are mentioned in Section S4 of ESI.† The experimental *J*–*V* parameters of the NiO-based devices at varying light intensities are used to validate our simulation model (see Fig. S13, ESI†). Fig. S14 (ESI†) shows that the *V*_OC_ starts decreasing significantly with the increase of interface trap states of concentration around 10^7^ cm^−2^. This further substantiates our experimental results as the simulated *V*_OC_ of NiO-based devices without any interfacial trap states is 1.08 V, while a decrease by more than 100 mV is simulated at an interfacial trap density of 10^10^ cm^−2^, matching the experimental *V*_OC_ of the NiO-based devices. The presence of traps and high recombination rate at the NiO/perovskite interface also affects the photocurrent as shown in Fig. 6. The total photocurrent is zero as this simulation is at open circuit condition. The electron and hole photocurrent without trap states are similar to the generation profile. On the other hand, a high electron and hole photocurrent with an inversion of its distribution is present at the NiO/perovskite interface with traps due to the high recombination leading to a decrease in hole concentration in that interface.^41^

**Fig. 7:**
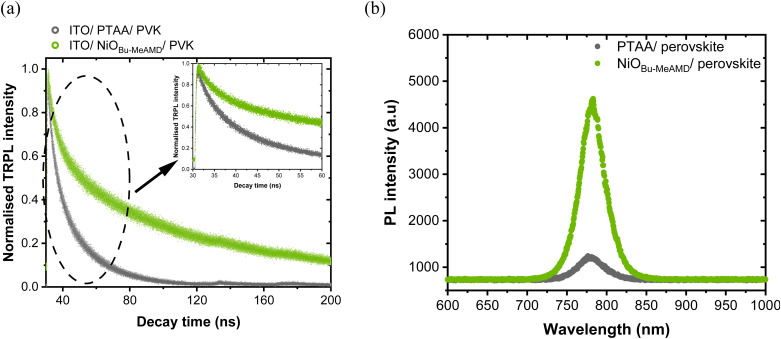
(a) TRPL and (b) PL of the partial stacks of glass/ITO/NiO_Bu-MeAMD_ or PTAA/perovskite. The inset in (a) shows the initial decay region to focus on the charge extraction by the HTL.

This leads us to conclude that high interface recombination accompanied by asymmetric band bending of the quasi-Fermi level impedes charge extraction at the NiO/perovskite interface and limits the *V*_OC_. Moreover, time-resolved PL (TRPL) in Fig. 7(a) supports this conclusion, as shown by the faster TRPL decay in the initial period of ∼50 ns for the PTAA-based stack indicating an enhanced hole extraction by PTAA as compared to NiO. Additionally, we observe a large quenching of the PL signal for PTAA (Fig. 7(b)) consistent with the TRPL results and the observed difference in *V*_OC_. Therefore, passivating the NiO/perovskite interface is crucial in suppressing the interfacial recombination.

**Fig. 8:**
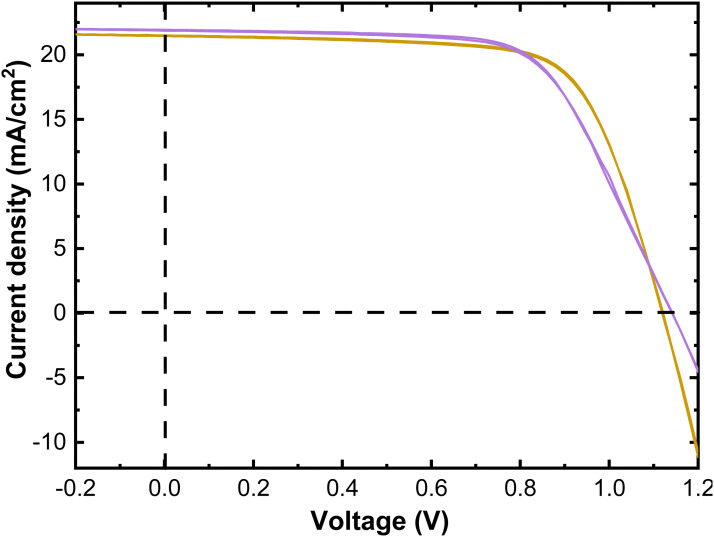
The forward and reverse scanned *J*–*V* curves of NiO_Bu-MeAMD_/Me-4PACz and Al:_NiOBu-MeAMD_/Me-4PACz-based devices after 3 minutes of light soaking.

Next, we investigate the passivation of NiO surface by Me-4PACz SAM. As concluded earlier, this approach may serve to disclose whether NiO resistivity affects the device performance, once the NiO/perovskite interface is passivated. The *J*–*V* parameters of the NiO-based devices, with and without Me-4PACz passivation are reported in Fig. 1. The *J*–*V* curves of the NiO/Me-4PACz-based devices after light soaking are presented in Fig. 8 and *J*–*V* parameters are summarised in Table 4. Light soaking for nearly 3 minutes is carried out because Al:NiO_Bu-MeAMD_/Me-4PACz-based devices exhibited a S-shaped *J*–*V* curve, likely due to charge transport barrier or band misalignment,^82^ which disappeared upon light soaking. Passivating the NiO_Bu-MeAMD_ and Al:NiO_Bu-MeAMD_ layers with Me-4PACz leads to the improvement of the *V*_OC_ by 170- 200 mV compared to the unpassivated devices (Table 3). The NiO_Bu-MeAMD_/Me-4PACz-based devices also have a *J*_SC_ gain of ∼1 mA cm^−2^ leading to a PCE of ∼17.5%. However, there is a fill factor loss of ∼4%, compared to the unpassivated device (Table 3), due to an increase in the *R*_series_ as shown in Table 4. We hypothesize that the increase in *R*_series_ is due to the formation of a carbazole-multilayer on NiO, instead of a monolayer.^83^ When comparing the Al:NiO_Bu-MeAMD_ with the highly resistive NiO_Bu-MeAMD_-based devices, after interface passivation, we observe that the *V*_OC_ and the *J*_SC_ are similar. So, in conclusion, the *V*_OC_ gain with respect to the unpassivated device is due to the trap passivation and better charge extraction by the Me-4PACz and is not further influenced by the NiO film resistivity. However, the FF of the Al:NiO_Bu-MeAMD_/Me-4PACz-based device is affected by the S-kink which could be potentially due to an increase in the valence band-offset to ∼0.5 eV between Me-4PACz (∼5.7 eV VBM^84^) and Al:NiO_Bu-MeAMD_ compared to the NiO_Bu-MeAMD_^85,86^ The FF increases from ∼54% to ∼64% due to the mitigation of the S-shape upon light soaking (see, Fig. S11, ESI†).^86^ Nevertheless, the Al:NiO_Bu-MeAMD_/Me-4PACz-based devices have a lower PCE compared to the NiO_Bu-MeAMD_/Me-4PACz-based devices due to the lower FF of the former devices as a consequence of a higher series resistance. Lastly, the processing of Me-4PACz and the perovskite on the NiO_MeCp_ layer did not lead to a full film coverage (image attached, see Fig. S12, ESI†) due to which further *J*–*V* measurements could not be carried out and hence it is not included here.

**Table 4:** Average values of PV parameters, shunt (*R*_shunt_) and series resistance (*R*_series_) along with their standard deviation of the NiO/Me-4PACz-based PSCs. The *R*_shunt_ and *R*_series_ are calculated from the slope of the *J*–*V* curve at the *J*_SC_ and *V*_OC_ respectively. This data is collected from several batches on 30 devices with PTAA and 15 devices with NiO/Me-4PACz

	V_OC_ (mV)	J_SC_ (mA cm^−2^)	FF (%)	PCE (%)	R_series_ (Ω cm^2^)	R_shunt_ (kΩ cm^2^)
PTAA	1064 ± 10	20.9 ± 0.6	79 ± 2	17.5 ± 0.7	3.7 ± 0.3	3.8 ± 1.2
NiO_Bu-MeAMD_/Me-4PACz	1131 ± 8	21.6 ± 0.3	72 ± 2	17.5 ± 0.7	7.7 ± 0.9	3.2 ± 1.2
Al:NiO_Bu-MeAMD_/Me-4PACz	1135 ± 6	21.4 ± 0.4	64 ± 2	15.7 ± 0.6	14.6 ± 1.9	1.8 ± 0.3

## 4. Conclusion

In summary, we evaluate the performance of NiO_Bu-MeAMD_, Al:NiO_Bu-MeAMD_ and NiO_MeCp_-based devices, compared to PTAA-based devices. The NiO_Bu-MeAMD_ is doped with aluminium using a novel ALD process developed in this work. Decreasing the resistivity of NiO, in case of Al:NiO_Bu-MeAMD_ and NiO_MeCp_, increases the *V*_OC_ by ∼30 mV with respect to the NiO_Bu-MeAMD_-based device. However, no improvement in device performance occurs because of a lower FF in the former devices. We also conclude that the performance of PTAA-based devices surpasses that of NiO-based devices primarily due to a 90–130 mV *V*_OC_ gain. Ideality factor of the NiO-based devices, which is in the range of 1–1.3, reveals that the NiO/perovskite interface is defective as compared to the PTAA/perovskite interface, resulting in *V*_OC_ loss. We do not consistently observe the PbI_2_ peak in the NiO-based partial stacks which is indicative of the interfacial redox reaction leading to a *V*_OC_ loss.

On the other hand, analysis of absolute photoluminescence measurements and the variation of FF with light intensity demonstrate that trap-assisted interfacial recombination is the cause behind the *V*_OC_ loss in NiO-based devices. A high rate of trap-assisted recombination at the NiO/perovskite interface can lead to band bending of the quasi-Fermi level for holes at the interface thereby decreasing the *V*_OC_. SCAPS simulation results further confirm that the presence of a high trap density at the NiO/perovskite interface results in a decrease in the *V*_OC_. Our work also shows that whilst lowering the NiO resistivity has a limited positive effect on the *V*_OC_ of the devices built only on NiO as HTL, modifying the NiO/perovskite interface with Me-4PACz SAM enabled a *V*_OC_ increase of 170–200 mV, showing that passivating this interface is key to the increase in device performance. Also, once the interface is passivated, the NiO resistivity has no further influence on the *V*_OC_ within the explored range of resistivity of around 70 to 1.7 × 10^4^ Ω cm. Moreover, we would like to highlight that it is necessary to compare the performance of NiO-based devices with a state-of-the-art HTL to draw conclusion on the efficacy of tuning a specific material property on device performance.

## Data availability

The data supporting this article have been included as part of the ESI.†

## Conflicts of interest

There are no conflicts to declare.

## Supplementary Material

MA-005-D4MA00873A-s001
