# Supplementary material for: On the VOC loss in NiO-based inverted metal halide perovskite solar cells
Source: Mater Adv. 2024 Oct 14;5(21):8652–64. doi: 10.1039/d4ma00873a (PMC11472218; doi:10.1039/d4ma00873a)
Supplement: MA-005-D4MA00873A-s001 [file MA-005-D4MA00873A-s001.pdf]

Supplementary Information (SI) for Materials Advances.
This journal is © The Royal Society of Chemistry 2024

Supplementary information

for

On the V_{OC} Loss in NiO-based Inverted Metal Halide Perovskite Solar Cells

Kousumi Mukherjee [a] , Denise Kreugel [a], Nga Phung [a], Cristian van Helvoirt [a], Valerio Zardetto [b], Mariadriana Creatore [a,c]

[a] Department of Applied Physics and Science Education, Eindhoven University of Technology, P.O. Box 513, 5600 MB Eindhoven, The Netherlands

[b] TNO Partner in Solliance, High Tech Campus 21, Eindhoven 5656 AE, The Netherlands

[c] Eindhoven Institute of Renewable Energy Systems (EIRES), PO Box 513, 5600 MB Eindhoven, The Netherlands

Section S1. Method

Process description for $NiO_{Bu-MeAMD}$

The $NiO_{Bu-MeAMD}$ process had been adapted from the recent work of Phung et al. N, N'-di-tert-butylacetamidinato)Nickel(II) (Ni(^{t}Bu-MeAMD)$_2$) from STREM chemicals (99.999% metal purity) and water are used as the precursor and co-reactant respectively. The Ni(^{t}Bu-MeAMD)$_2$ bubbler is kept at 90°C and an argon flow of 100 sccm through the bubbler is used for delivery at a temperature of 125°C. The chamber temperature is set at 120°C and the deposition is carried out at a table temperature of 150°C. One NiO ALD cycle consisted of 4 s Ni(^{t}Bu-MeAMD)$_2$ dose, 20 s precursor purge using Ar, 50 ms H_2O dose, and 60 s H_2O purge by Ar. A GPC of ~ 0.044 nm/cycle is observed at the saturation condition.

Process description for NiO_{MeCp}

The NiO_{MeCp} process had been adopted from the process developed by Koushik et al. consisting of a 4 s bis-methylcyclopentadienyl-nickel (Ni(MeCp)$_2$) (Sigma-Aldrich, 97% purity) precursor dose, followed by 3 s precursor purge using Ar, 3 s O_2 plasma co-reactant exposure and lastly, a 1 s Ar purge. The Ni(MeCp)$_2$ bubbler is kept at 55 °C and an argon carrier gas is used for the delivery via a delivery line heated to 75 °C.

Process development of Al: $NiO_{Bu-MeAMD}$

The ALD process of Al:$NiO_{Bu-MeAMD}$, developed in this work, is carried out using a supercycle approach. Here, '*m*' cycles of NiO are followed by the aluminium incorporation step and lastly end with another '*m*' cycles of NiO, as shown in Fig. S1. This is repeated for '*N*' (here, *N*=2) supercycles to get a film thickness of around 10-12 nm.

In this work, we have used dimethyl aluminium isopropoxide (DMAI) as the Al precursor instead of the commonly used trimethyl aluminium (TMA). TMA has a very high growth per cycle (GPC) of ~1.1 Å/cycle,[1] compared to NiO (~0.44 Å/cycle) making it hard to control the Al incorporation in the NiO film. Moreover, Baker et al. and Hossain et al. observed an enhanced growth of Al_2O_3 on NiO, using TMA, resulting in a Ni-deficient or Al-rich film.[2,3] On the other hand, the GPC of Al_2O_3 using DMAI is lower (~0.8 Å/cycle [1]) because the bulkier isopropoxide ligand provides steric hindrance to the chemisorption of the precursor, making it easier to control the incorporation of Al in the film.[4,5]

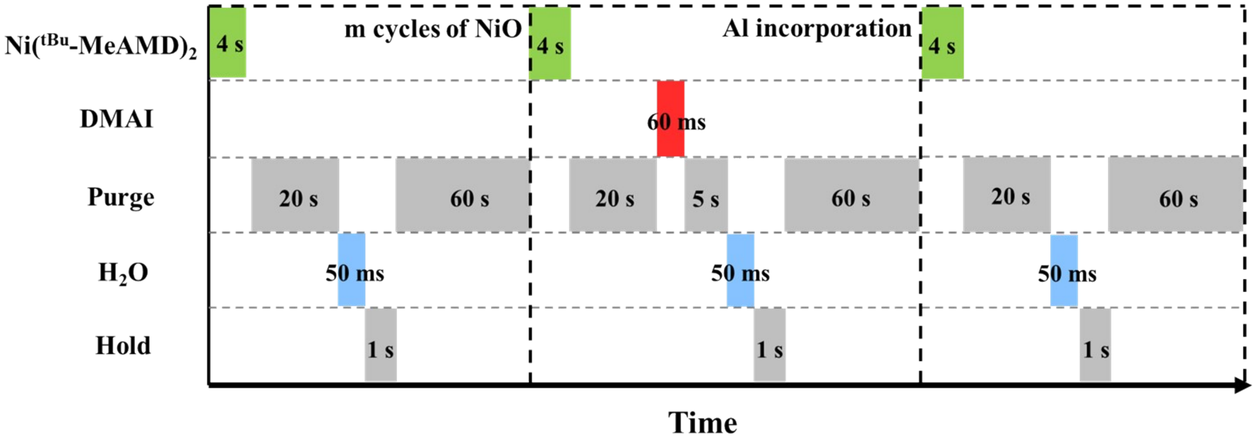

Figure S1. Schematic of supercycles of the Al: $NiO_{Bu-MeAMD}$ ALD process using sequential precursor dosing approach for the aluminium incorporation.

DMAI from Dockweiler Chemicals GmbH (≥99.9999 metal purity) is used here and the bubbler and delivery line temperature are kept at a temperature of 50°C and 70°C, respectively. The Al incorporation in the NiO film is carried out by the sequential precursor dosing strategy (*A*→*B*→*O*) consisting of a DMAI dose for 60 ms sequentially after Ni(^{t}Bu-MeAMD)$_2$ and it is followed by a 5 s Ar purge and a 50 ms H_2O water co-reactant step. The bulky ligands of Ni(^{t}Bu-MeAMD)$_2$ precursor block some of the active sites for chemisorption of DMAI which helps in further controlling the Al incorporation of the film.

The cycle ratio of NiO to Al_2O_3 ('*2m:1*') is varied to lower the resistivity of the doped NiO film as shown in Fig. . Doping NiO with Al creates shallow acceptor levels near the valence band maximum (VBM) reducing the resistivity.[6,2] The lowest resistivity is obtained for a NiO to Al cycle ratio of 135 and is used for the Al:$NiO_{Bu\text{-}MeAMD}$ HTL in this work.

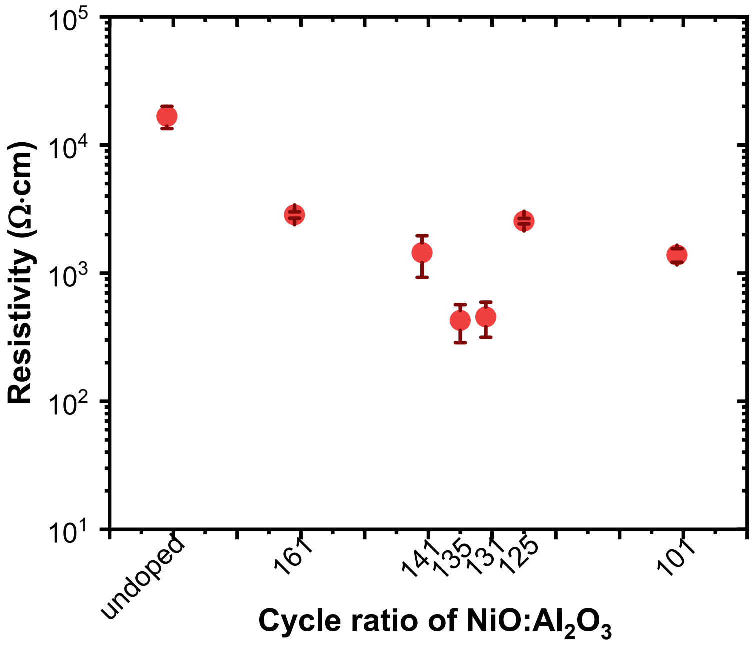

Figure S2. Resistivity variation as a function of the change in cycle ratio NiO:Al_2O_3.

Section S2. NiO characterization

Table S1. Elemental composition of $NiO_{Bu\text{-}MeAMD}$, $Al{:}NiO_{Bu\text{-}MeAMD}$ and NiO_{MeCp} as determined by RBS and ERD (for hydrogen). The carbon concentration is extracted from XPS and is expressed in terms of C/ (Ni+O+C) (%).

	**C at.%** *(from XPS)*	**Ni**	**O**	**Al**	**H**	**Ni:O**	**thickness (nm)**
		**(atoms/ nm_{geo}^{2})**					
		(from RBS)					
$NiO_{Bu\text{-}MeAMD}$	5 ± 1%	1009 ± 20	1071± 43	-	263 ± 8	0.94 ± 0.04	25 ± 1
$Al{:}NiO_{Bu\text{-}MeAMD}$	5 ± 1%	383 ± 8	435 ± 13	7.5 ± 0.3	141 ± 7	0.88 ± 0.03	~11
NiO_{MeCp}	~1%	1288 ± 26	1339± 50	-	184±18	0.96 ± 0.04	23 ± 1

A larger detector and a longer measurement time was used to detect the amount of Al dopant accurately. The ALD NiO films contain carbon and negligible nitrogen impurities in the bulk of the film. The carbon detected in all the NiO films by RBS is less than 100 atoms/ nm^2 and this quantification involves huge uncertainty. Depth profile from XPS reveals that the undoped and $Al{:}NiO_{Bu\text{-}MeAMD}$ films contain higher carbon impurity, nearly 5%, most certainly linked to the presence of unremoved ligands from the precursors.

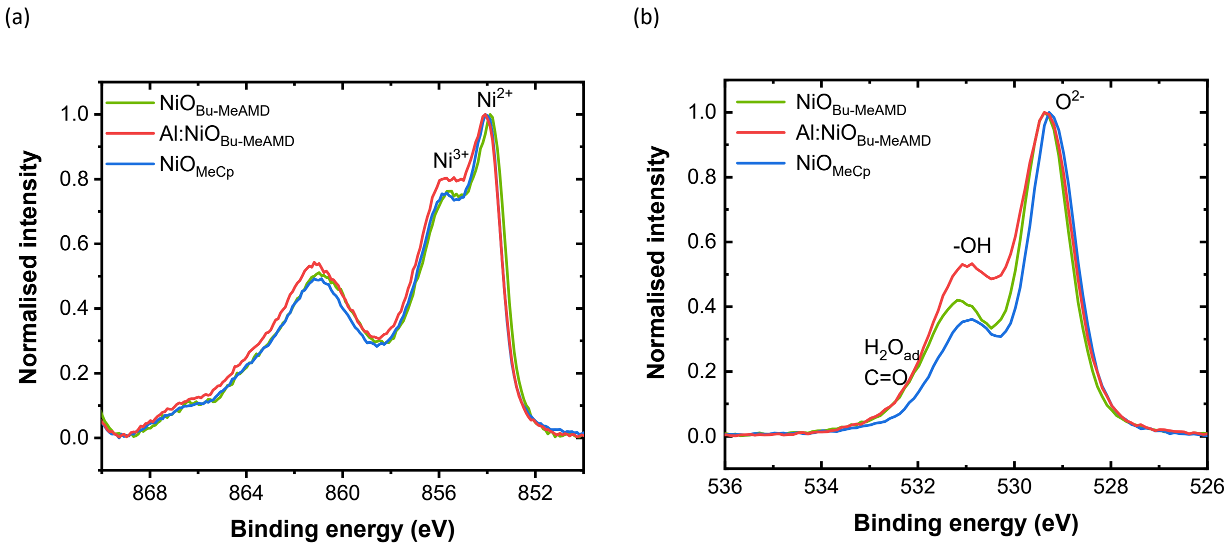

Figure S3. (a) Ni2p XPS spectra of $NiO_{Bu\text{-}MeAMD}$, $Al{:}NiO_{Bu\text{-}MeAMD}$ and NiO_{MeCp} normalised to the highest intensity. The main features of nickel in its +2 oxidation state is at 854 eV. The peak at 856 eV corresponds to nickel in its +3 state and non-local screening.[7] The broad features at higher binding energy (~861 eV, ~864 eV and ~866eV) are due to shake-up processes (satellites) of NiO, following the assignment reported in the literature.[8] (b) O 1s XPS spectra of $NiO_{Bu\text{-}MeAMD}$, $Al{:}NiO_{Bu\text{-}MeAMD}$ and NiO_{MeCp} normalised to the highest intensity. The main features at ~529 eV and ~531 eV indicate the presence of Ni-O bonds and the hydroxyl groups respectively.[9]

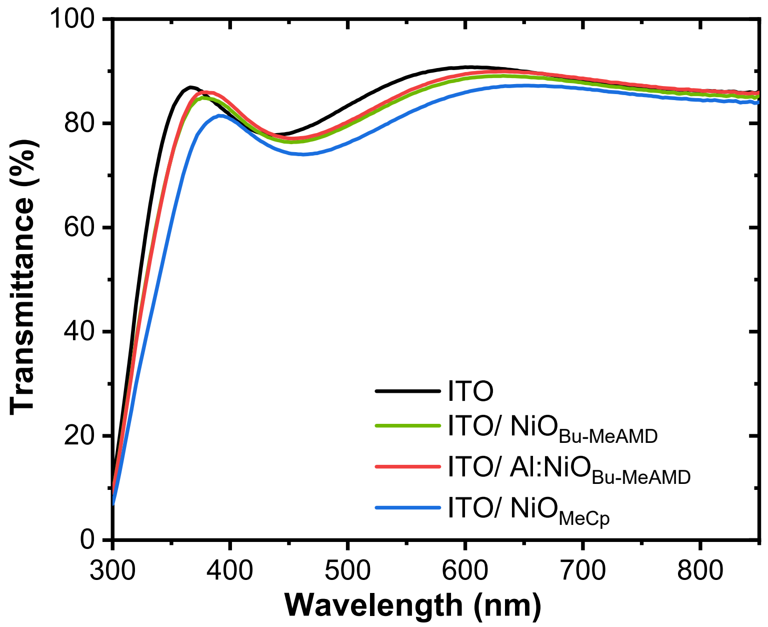

Figure S4. Transmittance of the 3 different NiO deposited on glass/ ITO substrates.

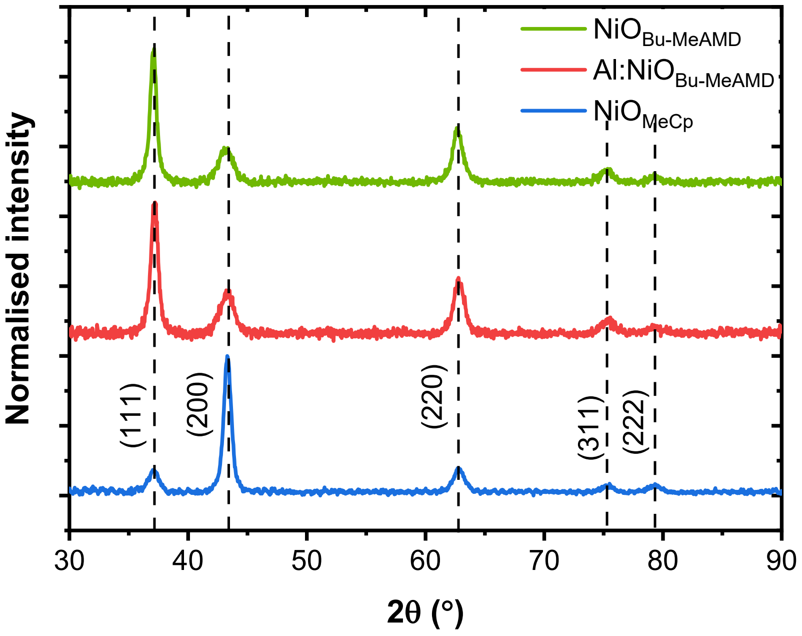

Figure S5. X-ray diffraction pattern of $NiO_{Bu\text{-}MeAMD}$, Al:$NiO_{Bu\text{-}MeAMD}$, and NiO_{MeCp}. All ALD NiO display a rock salt (face-centered cubic) crystallographic phase with peaks at around 37.1°, 43.3°, 62.8°, and 75.3° corresponding to the (111), (200), (220) and (311) crystal planes respectively.[10] No additional peaks are present due to the introduction of Al in the NiO lattice. The (111) orientation has alternating planes of O2- and Ni2+ ions resulting in a polar surface, whereas the (200) surface is non-polar due to the presence of both O2- and Ni2+ ions present alternating in the single plane.[11]

(a) (b)

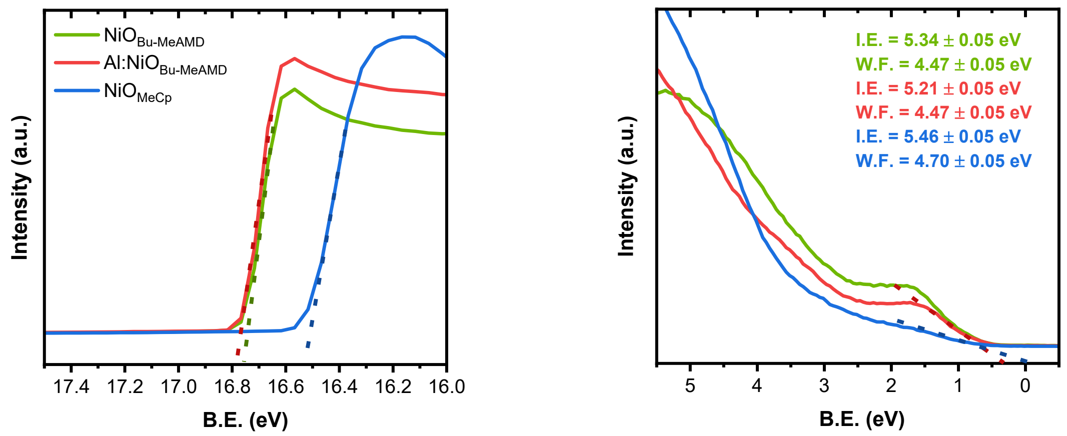

Figure S6(a-b). UPS spectra of $NiO_{Bu\text{-}MeAMD}$, Al:$NiO_{Bu\text{-}MeAMD}$ and PAALD NiO. The ionisation energy (I.E.) and work function (W.F.) of the different NiO are mentioned in the inset.

Charge carrier transport in NiO

As-deposited NiO primarily has Ni^{2+} vacancies (formation energy, ΔE_f = -0.19 eV).[12] The slightly non-stoichiometric O-rich or Ni-deficient films result in the formation of Ni^{3+}. The presence of excess oxygen or Ni vacancy (denoted as *'Ni_V'* in Kroger- Vink notation) in the lattice leads to the oxidation of two Ni^{2+} ions to Ni^{3+} ions to maintain electroneutrality, as shown in equation 1.[13,14,15] The Ni^{3+} ion acts as acceptor i.e. it can accept a 2p e^- from a nearby O^{2-} ion or a 3d e^- from an adjacent Ni^{2+} ion thereby creating a hole and resulting in the p-type conductivity of NiO.[16]

$$^{1}/_{2}O_2 + 2Ni^{2+} \rightarrow Ni_V + O^{2-} + 2\,Ni^{3+} \qquad (1)$$

Hence, an increase in the Ni^{3+}-to-Ni^{2+} ratio indicates an increase in the charge (hole) carrier concentration which in turn lowers the resistivity of NiO.[17] Creation of Ni^{3+} induces defect states in the band gap of NiO close to the valence band thereby decreasing the energy gap between the work function (WF) and the VBM (E_{VBM}-E_{WF}).[2]

This shows that the hole transport in NiO takes place from Ni^{3+} to Ni^{2+} sites. Therefore, the presence of Ni^{3+} sites at the interface doesn't help in charge extraction as holes are transferred to the Ni^{3+} sites.[18]

Section S3. Device characterization

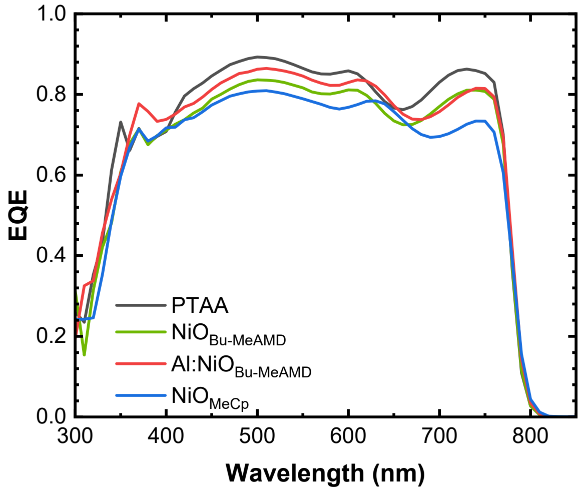

Figure S7. EQE spectra of the different HTL-based devices

Table S2. Comparison of the integrated current from the EQE spectra ($J_{SC,\ EQE}$) with the J_{SC} of the measured devices

	J_{SC} (mA/cm²)	Integrated $J_{SC,\ EQE}$ (mA/cm²)
PTAA	21.2	21.4
NiO$_{Bu\text{-}MeAMD}$	20.9	20.2
Al:NiO$_{Bu\text{-}MeAMD}$	21.4	20.8
NiO$_{MeCp}$	19.2	19.5

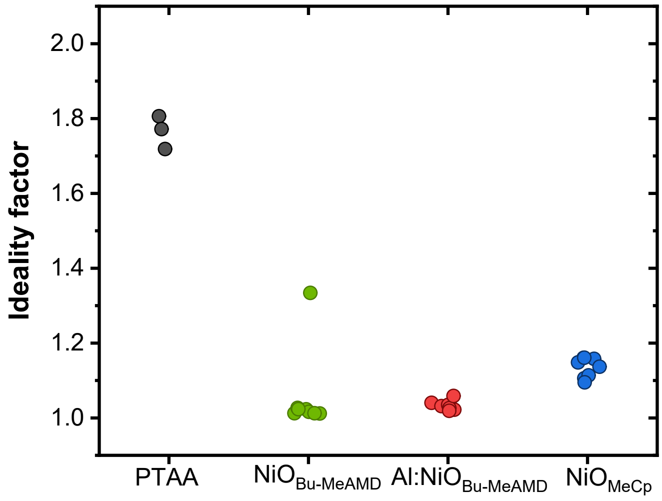

Figure S8. Ideality factor of different HTL-based devices (with PEAI passivating the ETL/ perovskite interface), calculated from the slope of the V_{OC} vs. light intensity graph.

(a)

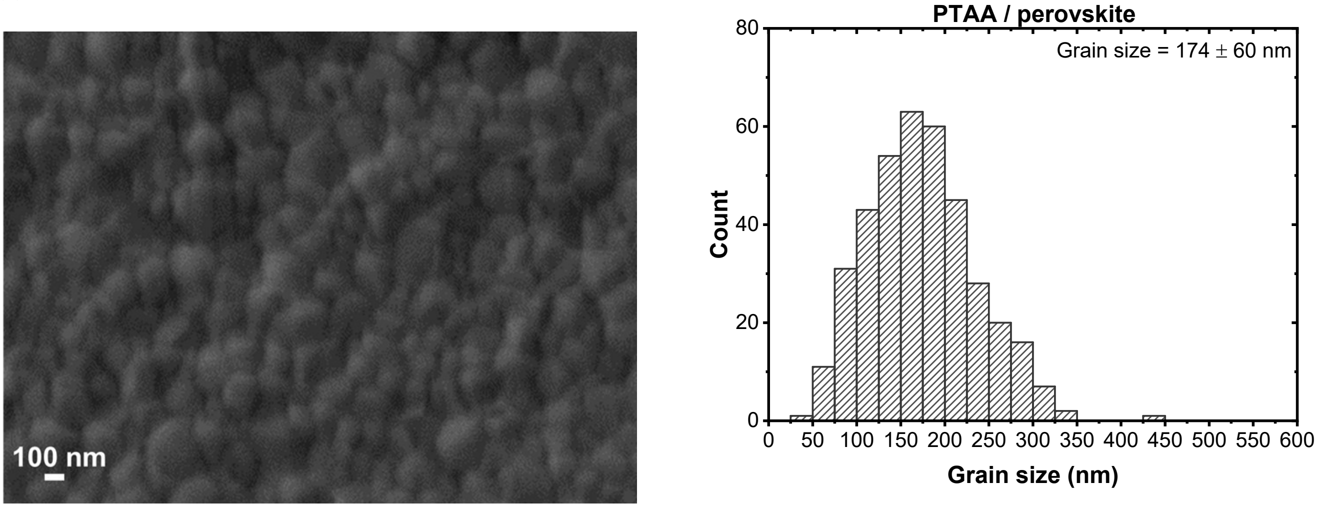
100 nm
PTAA / perovskite
Grain size = 174 ± 60 nm
Count
Grain size (nm)

(b)

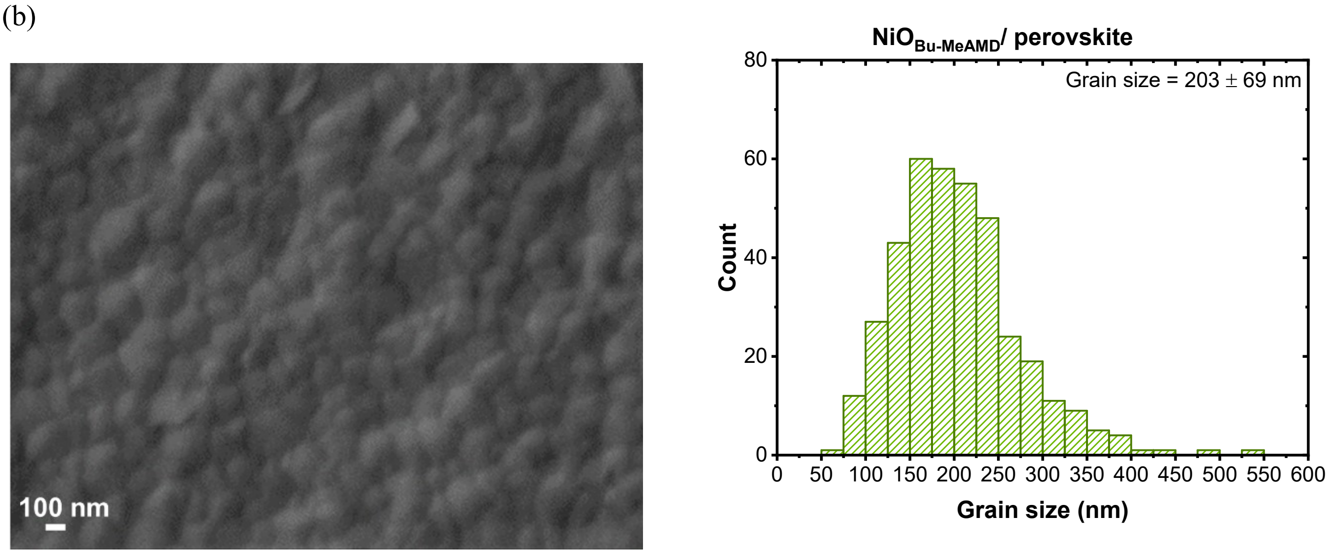
100 nm
NiO$_{Bu-MeAMD}$/ perovskite
Grain size = 203 ± 69 nm
Count
Grain size (nm)

(c)

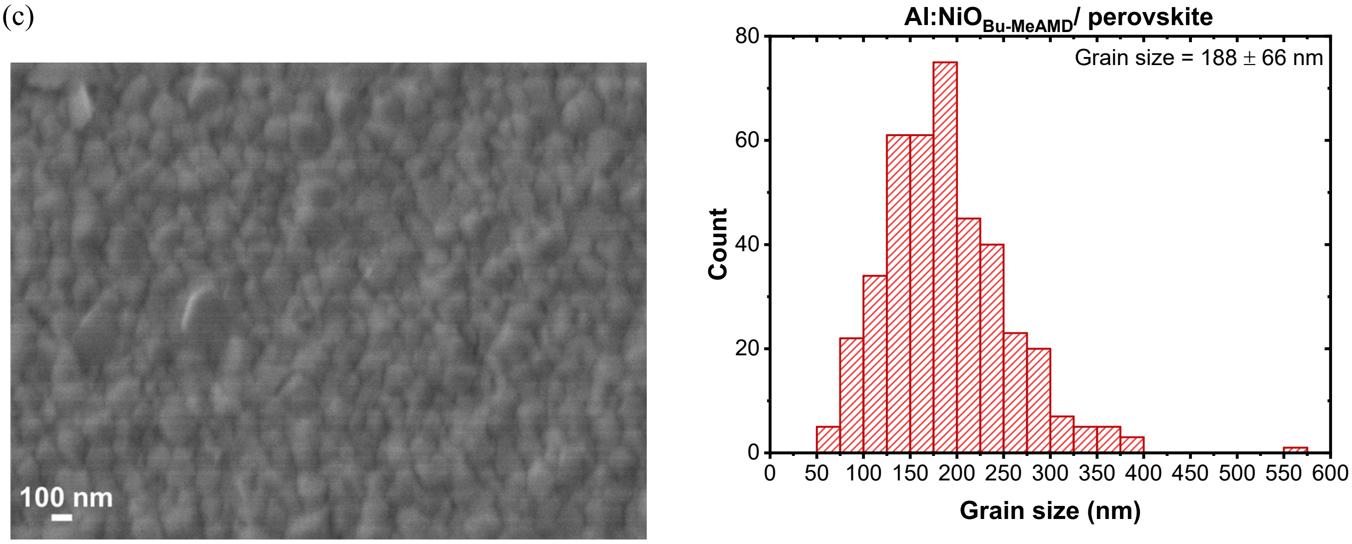
100 nm
Al:NiO$_{Bu-MeAMD}$/ perovskite
Grain size = 188 ± 66 nm
Count
Grain size (nm)

(d)

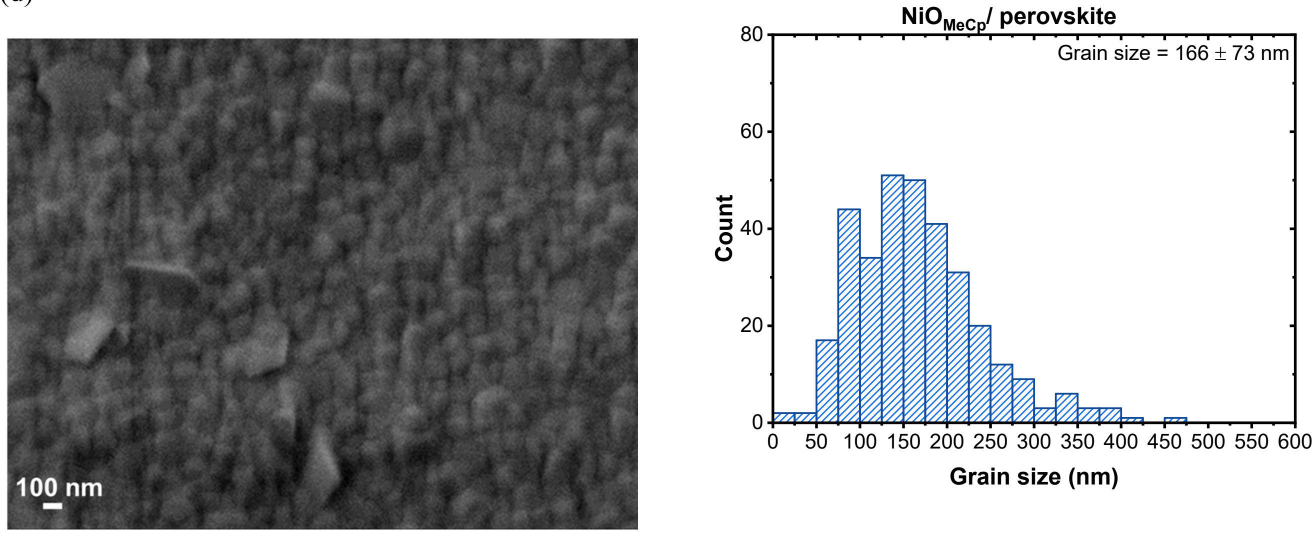

Figure S9. Scanning electron microscopy (SEM) images and grain size distribution of perovskite on (a) PTAA, (b) NiO$_{Bu\text{-}MeAMD}$, (c) Al:NiO$_{Bu\text{-}MeAMD}$, and (d) NiO$_{MeCp}$.

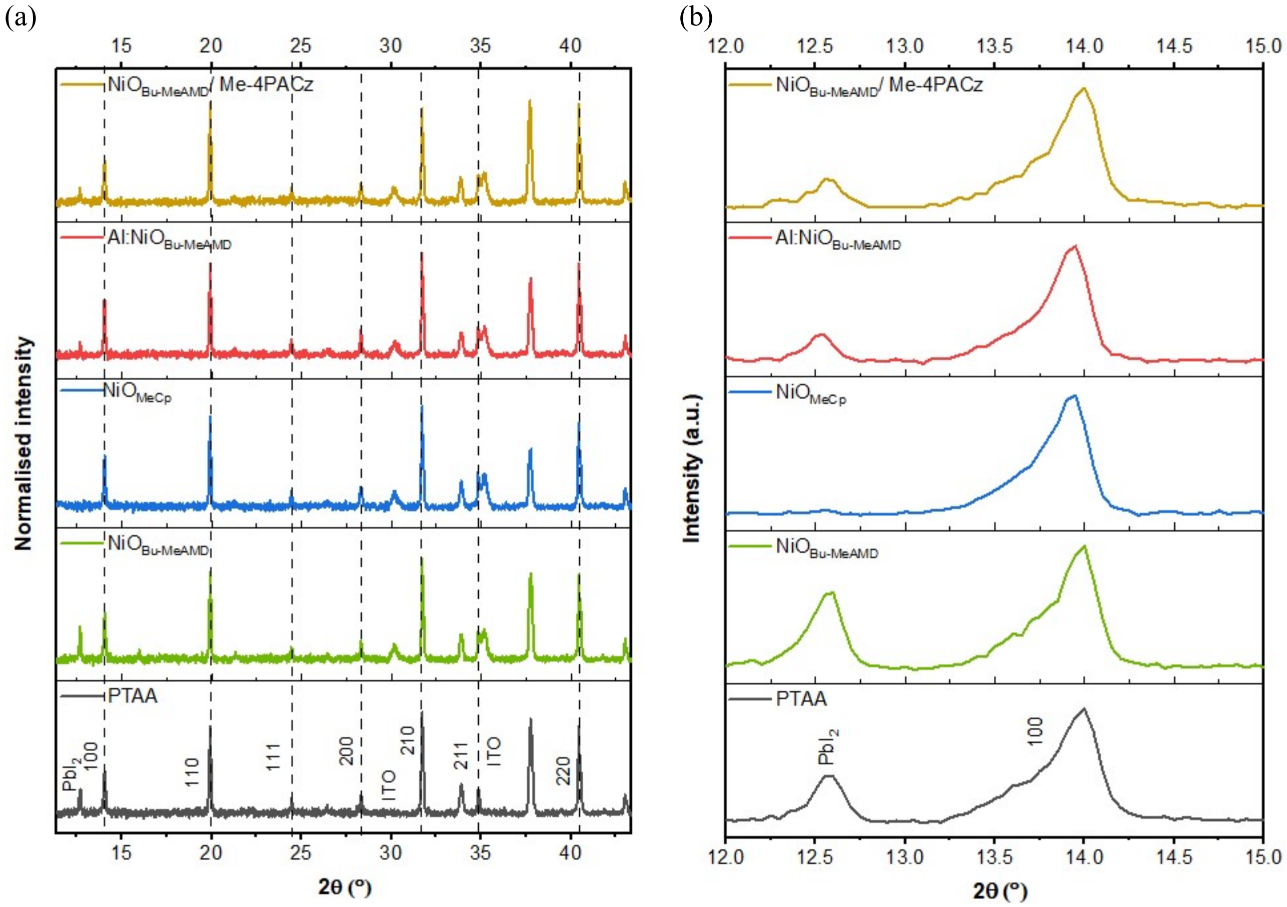

Figure S10. XRD pattern of perovskite on different HTL measured after 1 month by (a) Bragg- Brentano mode and (b) grazing incidence- XRD (GI-XRD) mode at an incidence angle of 1°.

The incidence angle of 1° for the GI-XRD measurement is chosen based on the penetration depth so that only the bulk of the perovskite is probed. The penetration depth of the X-ray is calculated from Lambert Beer's law using the energy of the X-ray used, attenuation coefficient and mass density of the perovskite. The mass density is calculated from the chemical composition of the perovskite and the lattice parameter as derived from the XRD pattern. The asymmetric peak broadening observed in the low-angle peaks is due to the axial divergence of the incident beam and is a measurement artefact. The presence of PbI_2 observed in this GI-XRD measurement indicates that it is primarily formed in the bulk of the perovskite due to degradation upon storage and exposure during the first measurement.

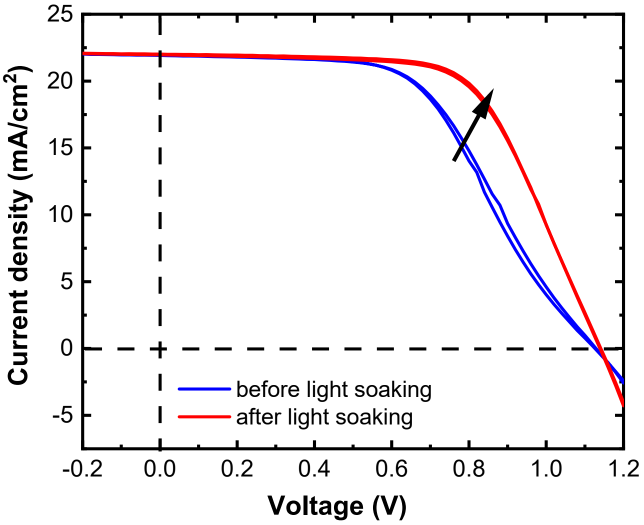

Figure S11. The forward and reverse scanned JV curves of Al:$NiO_{Bu\text{-}MeAMD}$/ Me-4PACz-based devices before and after light soaking showing the disappearance of the S-shape upon 3 minutes of light soaking.

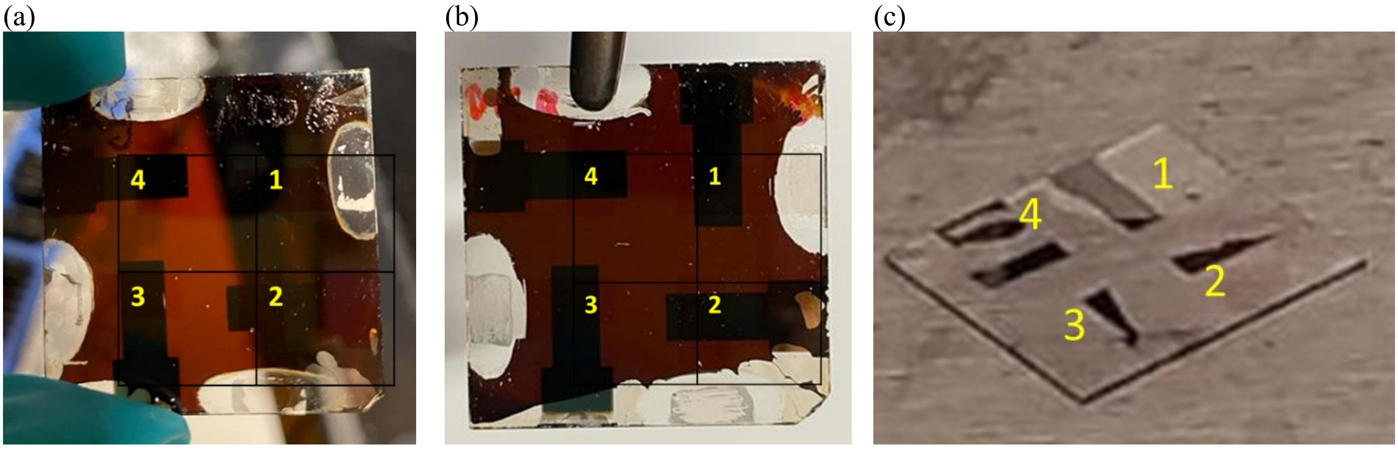

Figure S12. Images of perovskite processed on top of (a) NiO_{AMD}/ Me-4PACz, (b) Al:NiO_{AMD}/ Me-4PACz, and (c) NiO_{MeCp}/ Me-4PACz (substrate placed on hotplate). The pixels are labelled with numbers in yellow.

Section S4. SCAPS simulation of NiO HTL-based devices

Table S3. Parameters used for the HTL (NiO[19] and PTAA[20]), perovskite absorber [21,22] and ETL (PCBM[19,23] and BCP[23,24]) in the SCAPS simulation.

Parameter	NiO	Perovskite	PCBM	BCP
Thickness (nm)	11	450	40	5
Bandgap (eV)	3.3	1.57	2	3.5
Electron affinity (eV)	2.00	4.05	4.10	4.10
Relative dielectric permittivity	11.7	6.5	3.9	5.0
DOS_{CB} (cm^{-3})	$1.0*10^{20}$	$2.8*10^{19}$	$1*10^{21}$	$2.2*10^{21}$
DOS_{VB} (cm^{-3})	$2.8*10^{20}$	$3.9*10^{18}$	$1*10^{21}$	$1.8*10^{21}$
Electron mobility (cm^2/Vs)	$6*10^{-3}$	20	$1*10^{-2}$	$1*10^{-2}$
Hole mobility (cm^2/Vs)	$6*10^{-3}$	20	$1*10^{-2}$	$1*10^{-2}$
Uniform donor density (cm^{-3})	-	$1.3*10^{16}$	$5*10^{19}$	$1*10^{20}$
Uniform acceptor density (cm^{-3})	$3*10^{19}$	$1.3*10^{16}$	-	-

Both the acceptor and donor density in the perovskite absorber is $1.5*10^{16}$ cm^{-3}. The radiative recombination coefficient used for the charge carriers in the perovskite absorber is 10^{-9} cm^3/s. The effect of NiO/ perovskite trap density on the JV performance is found by varying it from 10^0 to 10^{15} cm^{-2}. The trap densities in the PCBM/ perovskite and NiO/ perovskite interface are 10^6 and 10^{10} cm^{-2} respectively.
The work function used for ITO (front contact) and copper (back contact) are 4.8 and 4.65 eV respectively. A pre-defined transmittance filter is used for the ITO.

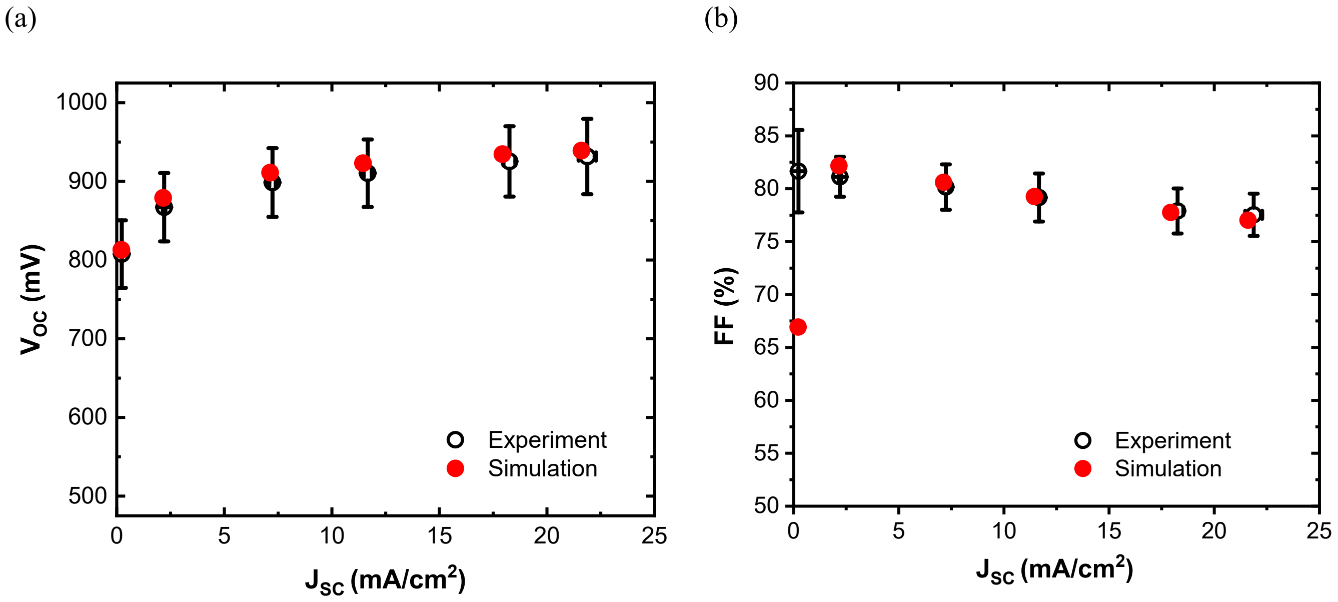

Figure S13. Experimental vs simulated (from SCAPS) JV parameters at different light intensity to validate the SCAPS model of the NiO-based PSC. The experimental data is based on the best performing devices from 2 different batches. An uniform acceptor type trap density of 10^{10} cm^{-2} is used for the NiO/ perovskite interface.

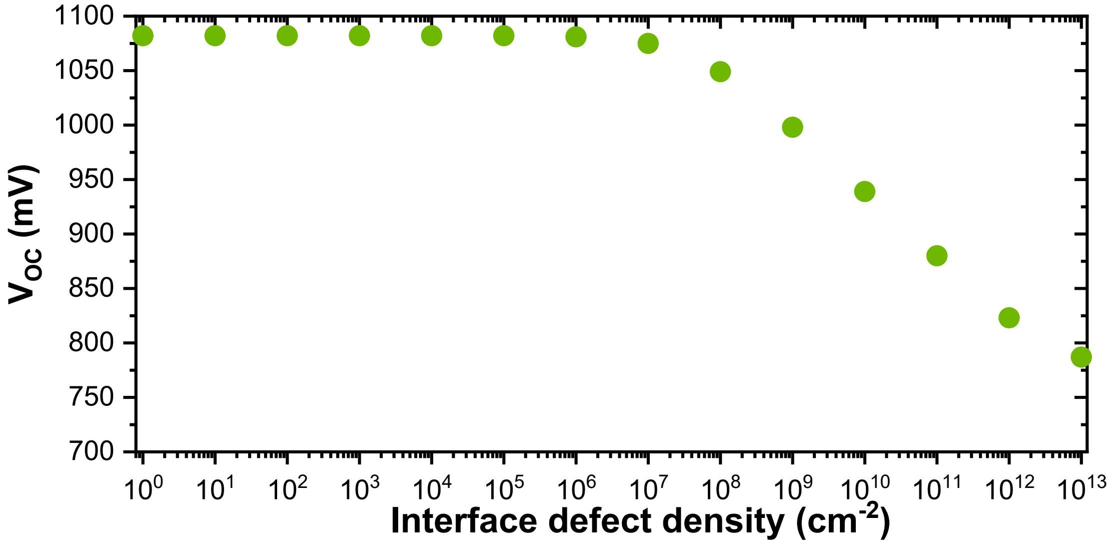

Figure S14. V_{OC} of the proposed device as a function of the defect density in the NiO/ perovskite interface.

References

1 S. E. Potts, G. Dingemans, C. Lachaud and W. M. M. Kessels, *Journal of Vacuum Science & Technology A*, 2012, **30**, 021505.
2 Md. A. Hossain, T. Zhang, Y. Zakaria, D. Lambert, P. Burr, S. Rashkeev, A. Abdallah and B. Hoex, *IEEE J. Photovoltaics*, 2021, **11**, 1176–1187.
3 J. G. Baker, J. R. Schneider, J. A. Raiford, C. de Paula and S. F. Bent, *Chem. Mater.*, 2020, **32**, 1925–1936.
4 S. Lee, M. Kim, G. Mun, J. Ko, H.-I. Yeom, G.-H. Lee, B. Shong and S.-H. K. Park, *ACS Appl. Mater. Interfaces*, 2021, **13**, 40134–40144.
5 Y. Wu, S. E. Potts, P. M. Hermkens, H. C. M. Knoops, F. Roozeboom and W. M. M. Kessels, *Chem. Mater.*, 2013, **25**, 4619–4622.
6 W. Zhang, H. Shen, J. Ge, B. Xu, P. Yan and J. Zhang, *J Mater Sci*, 2022, **57**, 15889–15900.
7 P. Nandi, H. Park, S. Shin, J.-W. Lee, J. Y. Kim, M. J. Ko, H. S. Jung, N.-G. Park and H. Shin, *Advanced Materials Interfaces*, 2024, **11**, 2300751.
8 R. T. M. van Limpt, M. Lavorenti, M. A. Verheijen, M. N. Tsampas and M. Creatore, *Journal of Vacuum Science & Technology A*, 2023, **41**, 032407.
9 N. Phung, M. Verheijen, A. Todinova, K. Datta, M. Verhage, A. Al-Ashouri, H. Köbler, X. Li, A. Abate, S. Albrecht and M. Creatore, *ACS Appl. Mater. Interfaces*, 2022, **14**, 2166–2176.
10 D. Koushik, M. Jošt, A. Dučinskas, C. Burgess, V. Zardetto, C. Weijtens, M. A. Verheijen, W. M. M. Kessels, S. Albrecht and M. Creatore, *Journal of Materials Chemistry C*, 2019, **7**, 12532–12543.
11 B. Liu, H. Yang, A. Wei, H. Zhao, L. Ning, C. Zhang and S. Liu, *Applied Catalysis B: Environmental*, 2015, **172–173**, 165–173.
12 J. Yu, K. M. Rosso and S. M. Bruemmer, *J. Phys. Chem. C*, 2012, **116**, 1948–1954.
13 D. Soo Kim and H. Chul Lee, *Journal of Applied Physics*, 2012, **112**, 034504.
14 K. O. Egbo, C. P. Liu, C. E. Ekuma and K. M. Yu, *Journal of Applied Physics*, 2020, **128**, 135705.
15 S. P. Mitoff, *J. Chem. Phys.*, 1961, **35**, 882–889.
16 V. Biju and M. Abdul Khadar, *Materials Research Bulletin*, 2001, **36**, 21–33.
17 H.-J. Seok, J.-H. Park, A. Yi, H. Lee, J. Kang, H. J. Kim and H.-K. Kim, *ACS Appl. Energy Mater.*, 2021, **4**, 5452–5465.
18 D. Di Girolamo, F. Di Giacomo, F. Matteocci, A. G. Marrani, D. Dini and A. Abate, *Chem. Sci.*, 2020, **11**, 7746–7759.
19 T. R. Lenka, A. C. Soibam, K. Dey, T. Maung and F. Lin, *CSIT*, 2020, **8**, 111–116.
20 J. Haddad, B. Krogmeier, B. Klingebiel, L. Krückemeier, S. Melhem, Z. Liu, J. Hüpkes, S. Mathur and T. Kirchartz, *Advanced Materials Interfaces*, 2020, **7**, 2000366.
21 S. Karthick, S. Velumani and J. Bouclé, *Solar Energy*, 2020, **205**, 349–357.
22 L. M. Herz, *ACS Energy Lett.*, 2017, **2**, 1539–1548.
23 S. A. Moiz and A. N. M. Alahmadi, *Polymers*, 2021, **13**, 2110.
24 N. Touafek, C. Dridi and R. Mahamdi, *J. Technol. Innov. Renew. Energy*, 2020, **9**, 1–6.
